# The Global Burden of Type 2 Diabetes Attributable to Dietary Risks Among Women: A 1990-2021 Analysis and Forecasting Through 2050

**DOI:** 10.2174/0115733998409251251105102446

**Published:** 2026-02-25

**Authors:** Tao Ding, Xiaoling Wang, Puwen Chen, Mengqi Zhong, Fuzhen Liu, Yu Xia

**Affiliations:** 1 Laboratory Medicine, First Affiliated Hospital of Gannan Medical University, Ganzhou, 34100, China;; 2 The First School of Clinical Medicine, Gannan Medical University, Ganzhou, 34100, China;; 3 School of Medical Technology, Gannan Medical University, Ganzhou, China

**Keywords:** Type 2 diabetes, mortality, disability-adjusted life years (DALYs), Global Burden of Disease (GBD), socio-demographic index (SDI), forecasting

## Abstract

**Introduction:**

Type 2 diabetes (T2D) is one of the major contributors to global mortality and disability. Diet and gender are key aspects of T2D, yet limited attention has been given to how their intersection contributes to the T2D disease burden. This study aims to assess the global burden of type 2 diabetes (T2D) attributable to dietary risks among women, using data from the Global Burden of Disease (GBD) 2021 study, and to explore regional and age-specific disparities over time.

**Method:**

We conducted a retrospective analysis using GBD 2021 data from 1990 to 2021 across 204 countries and territories. Outcomes included female deaths and disability-adjusted life years (DALYs) due to dietary risk-attributable T2D, stratified by age, region, and socio-demographic index (SDI). Future trends were projected using ARIMA and exponential smoothing models.

**Result:**

In 2021, 197,699 deaths and 9.4 million DALYs in women were attributed to dietary risks for T2D. The highest age-standardized mortality rate (ASMR) was observed in low SDI regions (8.26 per 100,000), while the highest age-standardized DALY rate (ASDR) occurred in Oceania (800.83 per 100,000). From 1990 to 2021, female deaths increased by 118.5% and DALYs by 183.4%, with a 4.9% decline in ASMR but a 34.0% rise in ASDR. Decomposition analysis showed that epidemiological changes (56.36%) were the main contributor to DALY increases. Projections suggest further DALY growth through 2050, especially in low-income regions.

**Discussion:**

These findings show that dietary risk-related T2D imposes a heavy burden on women globally, with significant differences across regions. Low SDI regions face higher mortality rates, while disability remains considerable in high-income regions, reflecting gaps in long-term care. These patterns suggest that nutrition transitions and healthcare inequalities contribute to the growing burden, indicating the need for dietary interventions adapted to local needs.

**Conclusion:**

The burden of dietary risk-attributable T2D among women has increased substantially, with marked regional and socio-economic differences. The results underscore the need for targeted, context-specific dietary interventions, particularly in settings facing limited healthcare access and rising nutrition-related risks.

## 1 INTRODUCTION

Type 2 diabetes (T2D) is a growing global health crisis, with over 500 million cases reported worldwide and projections exceeding 700 million by 2045 [1, 2]. Dietary factors are among the most modifiable drivers of T2D burden, yet global assessments often overlook sex-specific risks and regionally distinct dietary transitions [3, 4].

Women face unique vulnerabilities due to both biological mechanisms-such as hormonal fluctuations affecting insulin sensitivity-and sociocultural constraints, including unequal food allocation and health-seeking behaviors, particularly in low- and middle-income countries (LMICs) [5, 6]. Despite these disparities, many epidemiological models aggregate data by sex or prioritize high-income settings, failing to account for intersectional variation in exposure and outcome.

While the Global Burden of Disease (GBD) framework offers a standardized platform for risk assessment, prior studies have insufficiently addressed how dietary exposures and socioeconomic contexts interact with gender [7]. This has implications for global health equity, especially given that urbanization, population aging, and nutrition transition are accelerating across diverse settings.

To address these limitations, we used GBD 2021 data to comprehensively quantify the burden of T2D attributable to dietary risks among women between 1990 and 2021, analyze temporal trends and socioeconomic disparities using the Socio-Demographic Index (SDI), and project future trajectories of mortality and disability through 2050. By integrating demographic, geographic, and policy dimensions, our study provides new insights into the structural determinants shaping gender-specific T2D outcomes and highlights the urgency of developing context-sensitive, equitable prevention strategies.

## 2 METHODS

### 2.1 Overview and Methodological Details

We conducted a retrospective observational study using publicly available data from the Global Burden of Disease (GBD) 2021 database, which provides standardized estimates of mortality and disability-adjusted life years (DALYs) for 204 countries and territories from 1990 to 2021. The burden of type 2 diabetes (T2D) attributable to dietary risks was identified according to the GBD cause hierarchy, which ensures consistency in disease definitions and comparability across regions and time. The data sources, modeling strategies, and validation methods used in the GBD study are published elsewhere [8]. Dietary risks were defined based on the Global Burden of Disease (GBD) 2021 comparative risk assessment framework. This includes insufficient intake of protective dietary components that have been causally linked to T2D. Exposure levels were estimated at the population level using food balance sheets, household consumption data, and dietary surveys. These estimates were then compared to theoretical minimum risk exposure levels (TMRELs), and population-attributable fractions (PAFs) were calculated using relative risks from published meta-analyses. The estimated burden reflects the portion of T2D mortality and DALYs attributable to suboptimal diet in each country, SDI region, and age group [8, 9].

We extracted the following primary measures for T2D from 1990 to 2021: annual number of deaths and DALYs, age-standardized mortality rate (ASMR), age-standardized DALY rate (ASDR), and estimated annual percentage change (EAPC). GBD estimates incorporate vital registration data, disease surveillance systems, national surveys, and hospital records. EAPC provides the average annual rate of change over a specified period and is calculated using the following formula:

EAPC = (e^β - 1) × 100

where β is the slope of the natural logarithm of the ASR over time, derived from the linear regression model:

ln(ASR) = β × Year + α

Here, α is the intercept. A positive EAPC indicates an increasing trend, while a negative EAPC suggests a decline. Statistical significance was defined as 95% CIs not crossing zero [10].

The burden estimates were derived from the Global Burden of Disease Study 2021, which integrates data from vital registration systems, population-based surveys, disease registries, and administrative sources. All estimates are subjected to rigorous internal consistency checks and Bayesian meta-regression modeling *via* DisMod-MR 2.1 to ensure comparability across space and time. GBD quantifies uncertainty through 95% uncertainty intervals (UIs), derived from 1,000 draws of the posterior distribution for each measure. These procedures provide robust estimation of disease burden while accounting for data sparsity, reporting biases, and inter-source variability [11].

### 2.2 Socio-Demographic and Regional Analysis

To evaluate the impact of socio-demographic development, countries and regions were stratified based on their Socio-Demographic Index (SDI), a composite measure comprising the total fertility rate, mean years of education for individuals aged 15 years and older, and lag-distributed income per capita. The SDI provides a useful framework to assess socio-economic influences on health outcomes. We plotted regional ASMR and ASDR against SDI values to examine patterns in disease burden across different socio-demographic strata.

Additionally, we performed a basic regional analysis by comparing trends in T2D mortality and DALYs across the GBD regions. We used the hierarchical clustering analysis to identify patterns of T2D burden in GBD regions based on the similarity of their disease trends [12].

### 2.3 Decomposition Analysis

To determine the contributions of epidemiological changes, population growth, and aging to changes in mortality and DALYs, we conducted a decomposition analysis. This approach quantified the role of disease incidence, demographic expansion, and population aging in shaping the trends observed over time [13].

### 2.4 Predictive Modeling

We used autoregressive integrated moving average (ARIMA) and exponential smoothing (ES) models to forecast future trends in T2D mortality and DALYs from 2022 to 2050. Both models were based on historical data and included a 95% uncertainty interval (*UI*) to account for potential variations in future disease burden. ARIMA was chosen to capture short-term temporal dependencies, while ES provided robustness for long-term projections, ensuring balanced forecasting accuracy. Model selection was based on visual inspection of temporal trends and minimization of the Akaike Information Criterion (AIC). For ARIMA, differencing was employed to ensure stationarity, and optimal (p, d, q) parameters were selected based on the AIC and the Ljung-Box test. For ES, the additive form was applied to accommodate linear trend structures. Model performance was evaluated using root mean square error (RMSE) and mean absolute percentage error (MAPE), calculated on a training (80%) and validation (20%) split. Across all regions, RMSE values were <5.0 and MAPE <15%, indicating acceptable predictive accuracy for epidemiological forecasting. These procedures align with prior burden of disease modeling practices, ensuring that long-term trends are not overfitted to short-term fluctuations [14, 15].

### 2.5 Statistical Analysis

We performed statistical analyses using R software (version 4.4.2). Descriptive statistics for mortality and DALYs were summarized as means with 95% UIs derived from 1,000 Monte Carlo simulations. Hierarchical clustering was carried out using the hclust function in R, with Euclidean distance as the similarity measure and Ward’s method for clustering. Log-linear regression was used to calculate EAPC, and statistical significance was determined at *p* < 0.05.

## 3 RESULTS

### 3.1 The Global Disease Burden of Type 2 Diabetes Attributable to Dietary Risks Among Women in 2021

In 2021, there were 197,699 (95%*UI*: 39,636-324,534) global female deaths from dietary risk-attributable type 2 diabetes (T2D), with an age-standardized mortality rate (ASMR) of 4.25 per 100 000 (95%*UI*: 0.85-6.98), and 9,396,286 (95%*UI*: 2,069,187-15,612,534) disability-adjusted life years (DALYs), corresponding to an age-standardized DALY rate (ASDR) of 207.18 (95%*UI*: 45.75-344.06) (Tables **1** and **2**).

By age, deaths in the 25-29, 30-34, 35-39, 40-44, and 45-49 groups were 532 (95%*UI*: 123-860), 836 (95%*UI*: 204-1,353), 1,354 (95%*UI*: 307-2,175), 2,476 (95%*UI*: 519-3,988), and 5,256 (95%*UI*: 1,189-8,506), respectively; corresponding ASMRs ranged from 0.18 (95%*UI*: 0.04-0.30) to 2.23 (95%*UI*: 0.50-3.61). DALYs in these same age groups were 164,853 (95%*UI*: 43,516-290,062), 245,862 (95%*UI*: 60,284-428,279), 326,077 (95%*UI*: 77,774-553,780), 434,300 (95%*UI*: 102,590-743,738), and 644,611 (95%*UI*: 156,014-1,084,578), with ASDRs ranging from 56.65 (95%*UI*: 14.95-99.68) to 273.55 (95%*UI*: 66.21-460.26) (Tables **1** and **2**, Fig. **1**).

Across Socio-Demographic Index (SDI) regions, deaths ranged from 33,043 (95%*UI*: 7,316-52,395) in high SDI (ASMR 2.46, 95%*UI*: 0.56-3.86) to 59,594 (95%*UI*: 11,187-100,488) in middle SDI (ASMR 4.36, 95%*UI*: 0.81-7.36) and 46,687 (95%*UI*: 9,331-78,071) in low-middle SDI (ASMR 6.88, 95%*UI*: 1.35-11.55), while low SDI reported 18,450 (95%*UI*: 2,905-30,965) deaths but the highest ASMR (8.26, 95%*UI*: 1.28-14.01). DALYs ranged from 2,025,651 (95%*UI*: 503,351-3,353,452) in high SDI (ASDR 206.16, 95%*UI*: 52.39-338.37) to 777,284 (95%*UI*: 135,466-1,294,966) in low SDI (ASDR 276.75, 95%*UI*: 47.46-467.42) (Tables **1** and **2**, Fig. **1**).

Among 54 GBD regions, Asia (90,296 deaths), World Bank Lower Middle Income (74,450), and Basic Health System (70,967) had the highest number of deaths, whereas Oceania (798), Australasia (824), and Andean Latin America (1,393) reported the fewest; in terms of DALYs, Asia (4,484,326), Basic Health System (3,610,674), and World Bank Upper Middle Income (3,370,701) were highest, while Oceania (33,339), Australasia (37,712), and Andean Latin America (58,167) were lowest. By age-standardized rates, Oceania (24.13), Southern Sub-Saharan Africa (18.80), and Southern Africa (15.04) showed the highest mortality, whereas High-income Asia Pacific (0.67), East Asia (1.78), and the Western Pacific Region (1.87) were lowest; for DALYs, Oceania (800.83), Southern Sub-Saharan Africa (544.08), and Southern Africa (431.94) were highest, while East Asia (123.46), East Asia & Pacific - WB (130.90), and the Western Pacific Region (132.74) were lowest (Tables **1** and **2**, Fig. **1**).

At the country level, India (28,579), China (18,861), and the United States (12,172) had the most deaths, whereas San Marino (1), Niue (0), and Tokelau (0) had the fewest; Fiji (66.68), the Marshall Islands (58.32), and Kiribati (40.64) exhibited the highest ASMR, while Ukraine (0.97), Japan (0.39), and Singapore (0.39) were lowest. In terms of DALYs cases, China (1,267,470), India (1,120,385), and the United States (878,371) had the largest burdens, whereas Nauru (42), Niue (11), and Tokelau (6) reported the smallest; Fiji (1,933.06), the Marshall Islands (1,871.49), and Samoa (1,292.13) recorded the highest ASDR, while Peru (96.59), Indonesia (90.43), and the Democratic People's Republic of Korea (89.46) had the lowest (Tables **S1** and **S2**, Fig. **2**). Details of years of life lost (YLLs) and years lived with disability (YLDs) can be seen in the corresponding figures and supplementary materials for reference (Fig. **S1**). These findings reflect the modeled impact of dietary exposures as assessed by the GBD framework. In low and low-middle SDI regions, where the attributable T2D burden is highest, population diets tend to be characterized by low intake of fiber, whole grains, fruits, and vegetables, combined with rising consumption of sugar-sweetened beverages and processed foods. These dietary patterns align with the global nutrition transition and may explain the observed increase in both deaths and DALYs [16]. In contrast, high SDI regions exhibit relatively lower ASMRs, possibly reflecting better access to preventive care and healthier dietary environments. However, the rising DALYs in these areas suggest an increase in disease duration and complications, even in the context of improved survival.

### 3.2 Temporal Trends of Dietary Risk-Attributable T2D Among Women (1990-2021)

From 1990 to 2021, global female deaths from dietary risk-attributable type 2 diabetes (T2D) rose by 118.5%, while disability-adjusted life years (DALYs) increased by 183.4%. Over the same period, the age-standardized mortality rate (ASMR) declined by 4.9%, whereas the age-standardized DALY rate (ASDR) climbed by 34.0%. The estimated annual percentage change (EAPC) of the ASDR was -0.3, indicating a downward trend in mortality, whereas the EAPC of the corresponding DALY rate was 1.44 (Tables **1** and **2**, Fig. **S2**).

By age group, ASMR remained relatively stable in women aged 25-39 years, showed a slight increase in those aged 40-44 years, and reached its highest level in the 45-49 age group, demonstrating an initial decline followed by a subsequent rise. In contrast, the total death count steadily increased across all age categories, peaking in the 45-49 group. The ASDR exhibited an upward trend in every group, with the largest gains occurring in women aged 45-49 years and the lowest in those aged 25-29 years. DALYs escalated in all age groups, with the highest burden among those aged 45-49 years and the lowest among 25-29 year-olds (Fig. **S3**).

In high- and high-middle Socio-Demographic Index (SDI) regions, the ASMR showed a general decrease, despite an increase in absolute deaths. Middle SDI areas experienced relatively stable mortality rates but a continuous increase in death counts. In contrast, low and low-middle SDI regions exhibited upward trends in both ASMR and overall deaths, with the highest ASMR and smallest absolute death numbers observed in low SDI settings. The DALY burden increased in all SDI groups, particularly in low-middle and middle SDI regions, while the age-standardized DALY rate grew more modestly in high and high-middle SDI areas. Low SDI countries exhibited the smallest total DALYs yet the highest ASDR (Fig. **S3**)

At the GBD region level, hierarchical clustering indicated that Southern Sub-Saharan Africa, Southern Africa, Central Asia, Northern Africa, and parts of the Middle East displayed significant increases in both mortality and DALY rates, whereas certain high-income regions achieved notable reductions (Fig. **3**).

Regarding national trends, Singapore (-53.85%), Switzerland (-37.70%), and Belgium (-35.53%) registered the largest percentage drops in mortality between 1990 and 2021, whereas Guatemala (1315.22%), Qatar (660%), and Honduras (580%) recorded the most pronounced rises, likely influenced by low 1990 baselines or sampling fluctuations. The greatest positive EAPCs for mortality were seen in Mauritius (4.84), Lesotho (4.54), and Guatemala (4.48), while the most pronounced declines appeared in Singapore (-5.6), Rwanda (-3.4), and Cyprus (-3.36). With respect to DALYs, all countries showed increasing absolute numbers; the smallest percentage growth occurred in Rwanda (7.22%), Italy (10.33%), and Saint Vincent and the Grenadines (13.97%), whereas Qatar (1257.82%), Guatemala (1044.05%), and Kuwait (871.59%) reported the largest surges. Lesotho (4.78), Guatemala (4.6), and Morocco (4.39) had the highest positive EAPCs for DALYs, whereas the steepest declines were observed in Rwanda (-3.4), Ethiopia (-2.77), and Cyprus (-2.08). The trends of YLLs and YLDs can be observed in the corresponding figures and supplementary materials for reference (Fig. **S1**-**3**).

### 3.3 Decomposition Analysis

From 1990 to 2021, the global increase in DALYs due to dietary risk-related type 2 diabetes in women was 3,207,554.82, with significant contributions from low-middle and middle SDI regions (923,220.21 and 907,865.55, respectively). Epidemiological changes contributed the most to the overall increase in DALYs globally (56.36%), followed by population growth (41.06%) and aging (2.57%). In high SDI and high-middle regions, epidemiological changes were the major contributor (77.82%,75.03%), while in low SDI regions, population growth was the largest driver (70.9%). In low-middle SDI areas, both population growth and epidemiological changes contributed almost equally, with a slight dominance from population growth (48.88% *vs.* 48.98%) (Fig. **4**).

For deaths, the total increase was 11,435.62, with the main contribution from population growth (80.03%), while aging had a smaller effect (7.25%). In high SDI and high-middle SDI regions, the contribution of population growth was negative, reflecting improvements in disease prevention and control (-2,487.72% and -262.35%, respectively), whereas in low SDI regions and middle regions, population growth had the largest contribution (112.69%,88.04%). Epidemiological changes contributed negatively in some areas and positively in others, such as high SDI regions and high-middle regions (2,489.89%,360.3%) (Fig. **4**).

### 3.4 Forecasting the Future Burden of Dietary Risk-Attributable T2D Among Women (2022-2050)

For the prediction (2022-2050), both the ARIMA and ES models forecast continued growth in the global burden of type 2 diabetes in women due to dietary risks. DALYs are projected to increase year by year, with the age-standardized DALY rate (ASDR) showing a steady upward trend. Deaths will also continue to rise, but with a slight decline in the age-standardized mortality rate (ASMR) according to the ARIMA model. The ES model predicts a slower increase in the age-standardized DALY rate (ASR of DALYs), with total deaths rising in parallel, while the age-standardized death rate (ASDR) is expected to decline gradually over the period (Fig. **5**).

## 4 DISCUSSION

The present study reveals a substantial global burden of type 2 diabetes (T2D) attributable to dietary risks among women, with over 197,000 deaths and 9.4 million DALYs reported in 2021. Disparities across Socio-demographic Index (SDI) regions underscore systemic inequities in dietary patterns and healthcare access. Low-SDI regions exhibited the highest age-standardized mortality rates (ASMR), despite lower absolute death counts, suggesting limited preventive care and delayed diagnosis may amplify case fatality [17, 18]. Concurrently, the DALY burden in high-SDI regions remained significant, indicating that although advanced healthcare systems reduce mortality, they may fail to address long-term disability and chronic complications [19]. These findings support and extend the epidemiological transition theory, highlighting that disease patterns evolve not only with aging but also with shifting nutrition environments and policy gaps [20].

Age-specific trends revealed a pronounced burden among women aged 45-49 years, with the highest ASMR and DALYs. This aligns with evidence suggesting that menopause-related metabolic changes-such as estrogen decline and increased visceral adiposity-impair insulin sensitivity and accelerate type 2 diabetes progression in midlife women [21]. The notable burden among younger women (25-29 years) signals a concerning trend towards the early onset of T2D-related disability. Recent cohort studies show rising diabetes incidence in adolescents and young adults globally, driven by shifts in ultra-processed food consumption and sedentary behaviors, which indicates that obesogenic environments are accelerating disease onset in early adulthood [22, 23]. Despite these developments, current prevention programs remain predominantly focused on older age groups, overlooking critical windows in young adulthood where life-course interventions could yield significant public health impact.

Geographically, the burden was concentrated in Asia and lower-middle-income regions, likely reflecting large population sizes and rapid dietary transitions. However, after age-standardization, Oceania and Southern Sub-Saharan Africa emerged with the highest rates, implicating additional drivers, such as undernutrition, food insecurity, and genetic predisposition. Recent critiques emphasize that global dietary guidelines often overlook local food environment variations, undermining their relevance and effectiveness in diverse settings [24]. Notably, although this study was based on GBD data at global and regional levels, the populations analyzed differ significantly in terms of dietary culture and food availability, which may influence both dietary exposures and susceptibility to type 2 diabetes. While we discussed possible dietary influences on the observed burden, these associations were not directly assessed within each population group. Dietary transitions vary widely between regions; for example, urban populations in Latin America may consume high levels of packaged snacks and sweetened beverages [25], whereas rural communities in South Asia may face undernutrition or limited dietary diversity [26]. Such heterogeneity calls for future research incorporating localized dietary assessments and culturally specific exposure data to improve the interpretability of burden estimates.

Between 1990 and 2021, female deaths due to dietary risk-attributable T2D nearly doubled, while DALYs rose by over 180%. Despite slight improvements in age-standardized mortality, the age-standardized DALY rate (ASDR) increased, suggesting longer disease duration and inadequate management of complications. These divergent trajectories highlight limitations in current prevention frameworks, which focus heavily on mortality and underemphasize chronic disability-particularly in women [27]. Although dietary factors are a leading modifiable risk for T2D, other interacting exposures-such as sedentary behavior, stress, and gender-based healthcare barriers-remain poorly integrated into burden estimation models. Studies have shown that women in LMICs often delay diagnosis due to caregiving roles and limited autonomy in food choice [28, 29]. Thus, future risk assessments should consider compound exposures, particularly in settings where dietary risks co-occur with social disadvantage.

Age-specific trends further highlight systemic vulnerabilities. Women aged 45-49 years bore the highest burden, with ASDR rising to 273.55 per 100,000 by 2021. This peak coincides with the intersection of prolonged exposure to poor dietary habits, declining estrogen levels, and cumulative metabolic damage-a phenomenon termed the “midlife metabolic crisis” in women [30]. Existing T2D prevention programs rarely target this age group specifically; instead, they prioritize older adults or the general population. The stability of ASMR in younger cohorts (25-39 years) may reflect delayed disease onset due to improved early-life nutrition in some regions, though this hypothesis requires validation through longitudinal cohort studies [31].

Socioeconomic stratification reveals stark contrasts in trajectory. High and high-middle SDI regions achieved ASMR reductions through integrated care models combining dietary guidelines, glycemic monitoring, and subsidized health services [32]. Low SDI regions saw ASMR increases, driven by unregulated food environments and inadequate chronic disease management [33]. These disparities align with the “nutrition transition” theory, where globalization accelerates the adoption of processed diets in low-income settings before infrastructure for disease prevention is established [34]. A 2020 study in India found that women in urban slums consumed more ultra-processed foods than rural counterparts, correlating with higher diabetes risk [35]. Despite this, regional policies lag behind global trade agreements, allowing unchecked promotion of unhealthy products.

Country-specific patterns reveal the critical role of governance and health policy. For example, Singapore’s sugar-sweetened beverage labeling initiative has shown measurable effects on reducing sugary drink consumption, while countries like Guatemala and Qatar continue to face rising Type 2 diabetes (T2D) mortality amid weak food policy enforcement [36]. These findings support calls for integrated public health strategies combining fiscal, regulatory, and educational interventions. The rise in DALYs across all SDI levels suggests a global failure to contain T2D disability. In high-income regions, prolonged survival with comorbidities-such as neuropathy and nephropathy-increases the disability burden despite medical advances [27]. Gendered healthcare access disparities may further exacerbate outcomes for women, as documented by delayed diagnosis and systemic bias in care delivery [37].

The gender-specific burden observed in this study may be partially explained by both biological and sociocultural factors. Biologically, particularly during perimenopause, women experience significant hormonal changes, including declining estrogen levels, which impair insulin sensitivity and fat distribution, contributing to higher T2D risk [38]. Visceral adiposity and reduced skeletal muscle mass during midlife further exacerbate metabolic vulnerability. In addition, sex differences in inflammatory responses and mitochondrial function have been implicated in differential glycemic control and diabetes progression between men and women [39]. Socioculturally, women in many regions face reduced autonomy over food choices, lower physical activity levels due to caregiving roles or social norms, and limited access to timely healthcare, especially in LMICs [40]. These intersecting barriers amplify disease burden and delay diagnosis, contributing to both higher DALYs and prolonged disease duration in women. Targeted interventions addressing these structural and biological determinants are urgently needed to reduce gender-based disparities in diabetes outcomes.

Decomposition analysis revealed socioeconomic stratification in T2D burden drivers (1990-2021), challenging traditional health transition paradigms. Globally, epidemiological changes-primarily shifts in dietary patterns and disease management-accounted for 56.36% of the rise in disability-adjusted life years (DALYs), surpassing the contributions of population growth and aging. As previously discussed regarding middle- and low-middle SDI regions, this dominance of epidemiological factors underscores the escalating impact of ongoing nutritional shifts, especially in rapidly developing contexts, where urbanization and globalization continue to drive the adoption of energy-dense diets high in refined sugars and saturated fats [41]. In South Asia, the replacement of traditional whole grains with energy-dense convenience foods has been linked to β-cell dysfunction and insulin resistance; yet, policy responses remain siloed within healthcare systems, rather than targeting food environments [42].

Striking disparities emerge when examining SDI- specific contributions. In high-SDI regions, epidemiological changes drove 77.82% of DALYs growth, reflecting paradoxical trends: improved survival rates from advanced diabetes care coexist with rising disability due to prolonged disease duration and comorbidities. Conversely, in low-SDI settings, population growth accounted for 70.9% of DALYs increases, highlighting the collision between demographic expansion and stagnant healthcare infrastructure. In Sub-Saharan Africa, high fertility rates and limited prenatal nutrition programs exacerbate intergenerational T2D risks, mirroring challenges in Latin American indigenous communities, where maternal undernutrition coexists with rising adolescent diabetes rates [43, 44]. The near-equal contributions of population growth and epidemiological shifts in low-middle SDI regions, such as North Africa, suggest a critical transition phase in which dietary risks and demographic pressures amplify one another-a phenomenon underexplored in current transition models [45].

For mortality, the global dominance of population growth masks divergent regional narratives. In high-SDI regions, population growth contributed negatively to death counts, a testament to successful prevention strategies such as mass screening programs and public health campaigns promoting Mediterranean-style diets [46]. The negative contribution of epidemiological changes to mortality in low-SDI regions warrants scrutiny. This finding likely reflects systemic underreporting in low-SDI regions, compounded by fragmented vital registration systems [47]. The starkly positive epidemiological contributions in high-SDI areas likely signal unintended consequences of industrialized food systems, such as the proliferation of ultra-processed “diet” products marketed as healthy but laden with artificial sweeteners, which may dysregulate glucose metabolism [48].

The projected trajectories of type 2 diabetes (T2D) burden attributable to dietary risks among women globally from 2022 to 2050 reveal a persistent rise in disability-adjusted life years (DALYs) alongside modest declines in age-standardized mortality rates (ASMR). The slower ASDR increase in the ES model may reflect gradual adoption of preventive measures, such as public health campaigns promoting dietary diversity, whereas ARIMA’s linear trajectory implies sustained failures to regulate obesogenic food environments [49]. This discrepancy underscores the need for adaptive models that integrate dynamic policy scenarios, such as global implementation of fiscal policies or stricter food-labeling regulations-interventions with heterogeneous efficacy due to varying enforcement capacities and cultural contexts [50]. While some nations have successfully reduced sugary beverage consumption through taxation, others face challenges from transnational corporate influence and informal market dynamics [51]. The projected decline in ASMR masks a critical paradox: while advanced diabetes care in high-resource settings has improved survival rates, it has also led to increased disability burdens due to prolonged disease duration. Low-resource regions continue to face premature mortality due to limited access to diagnostics and essential therapies like insulin, highlighting persistent healthcare inequities [52].

## 5 LIMITATIONS

This study has several limitations. The accuracy of mortality and disability data in low-SDI regions may be compromised by underreporting and incomplete vital registration systems, potentially underestimating true burdens. The decomposition analysis assumes linear interactions between population growth, aging, and epidemiological changes, which may oversimplify dynamic sociopolitical factors, such as conflict-induced food shortages or rapid urbanization. Additionally, the predictive models (ARIMA and exponential smoothing) do not account for future policy shifts or climate-related disruptions to food systems, limiting their utility in rapidly changing environments. While the study highlights gendered dietary risks, it does not explore intersectional factors, such as race or rural-urban divides, which may further stratify outcomes.

Although this study primarily focused on dietary risks, other modifiable and non-modifiable factors, such as genetic predisposition, insulin resistance traits, physical inactivity, and early-life exposures, may confound the observed associations. The GBD comparative risk assessment framework does not explicitly model genetic susceptibility or physical activity in dietary risk estimates, which may limit causal inference and burden attribution accuracy in certain populations. Finally, reliance on aggregate data obscures individual-level heterogeneity in dietary behaviors and metabolic responses, necessitating complementary cohort studies.

## CONCLUSION

The global burden of T2D attributable to dietary risks in women has risen markedly over the past three decades and is projected to continue increasing, especially in low-SDI regions. Although age-standardized mortality has declined slightly, the steady rise in DALYs highlights persistent disability and unmet preventive needs. These findings call for pragmatic, evidence-informed strategies tailored to regional contexts-such as improving access to affordable, healthy foods, implementing gender-sensitive dietary policies, and strengthening the delivery of preventive care. Further research should prioritize the evaluation of localized interventions and the refinement of predictive models that integrate both nutritional and social determinants.

## Figures and Tables

**Fig. (1):**
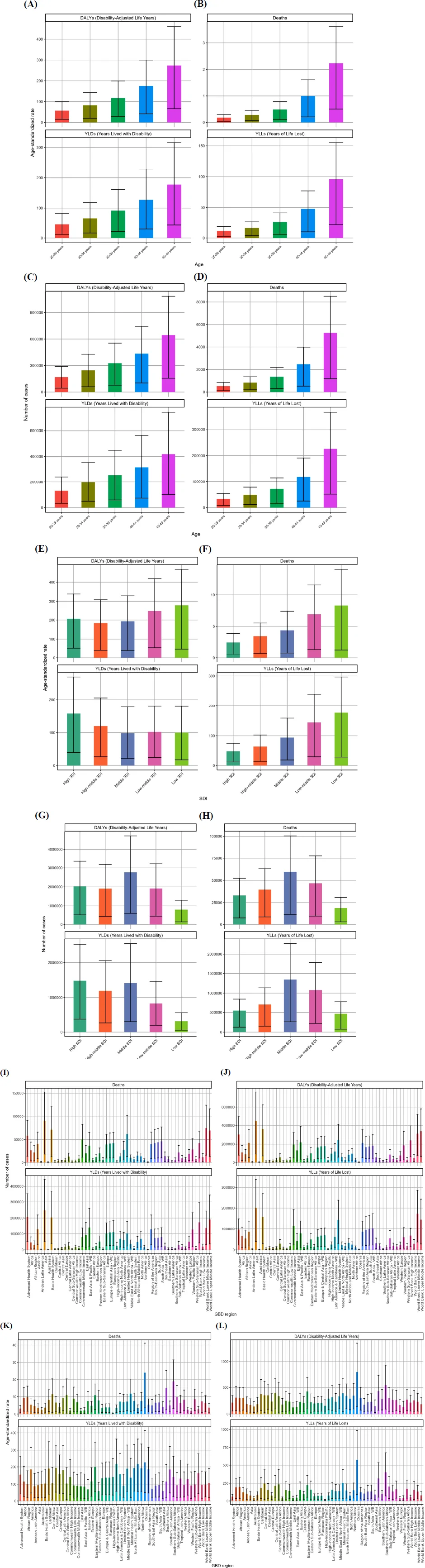
Numbers and age-standardized rates of dietary risk-attributable deaths, DALYs, YLLs, and YLDs among women in 2021. (**A**-**D**) Stratified by age group. (**E**-**H**) Stratified by Socio-Demographic Index (SDI) region. (**I**-**L**) Stratified by Global Burden of Disease (GBD) region. **Abbreviations:** DALYs, disability-adjusted life years; YLLs, years of life lost; YLDs, years lived with disability; SDI, Socio-Demographic Index; GBD, Global Burden of Disease.

**Fig. (2):**
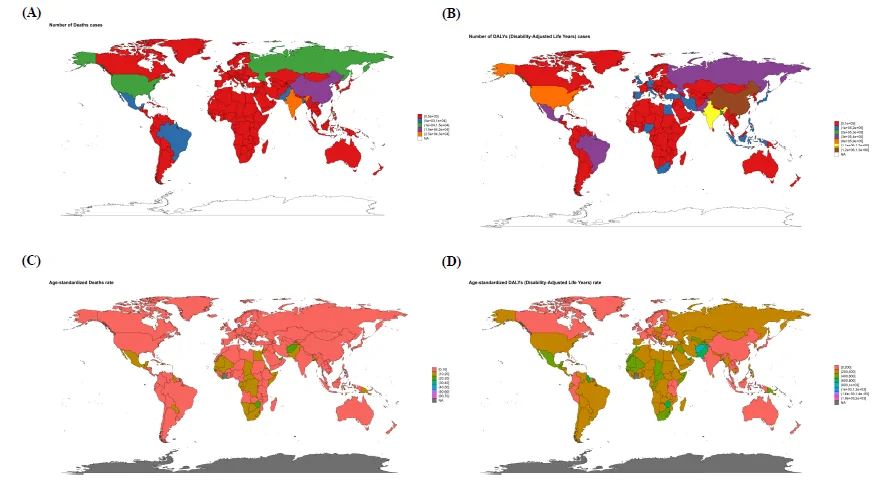
Global distribution of dietary risk-attributable deaths and DALYs among women in 2021. (**A**) Number of deaths; (**B**) Age-standardized death rate per 100,000 population; (**C**) Number of DALYs; (**D**) Age-standardized DALY rate per 100,000 population. **Abbreviations:** DALYs, disability-adjusted life years.

**Fig. (3):**
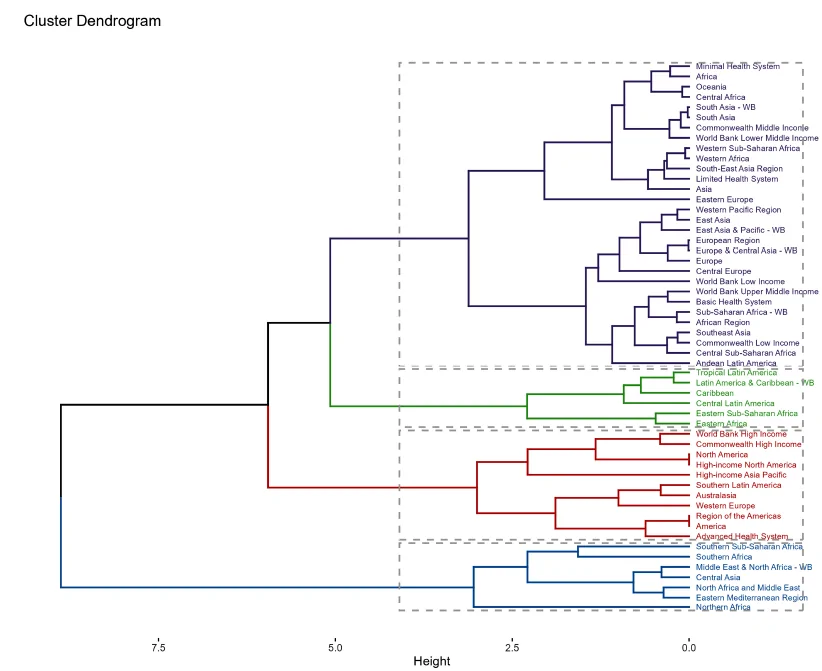
Results of cluster analysis based on the EAPC values of dietary risk-attributable age-standardized rates for deaths and DALYs from 1990 to 2021. **Abbreviations:** EAPC, estimated annual percentage change; DALYs, disability-adjusted life years.

**Fig. (4):**
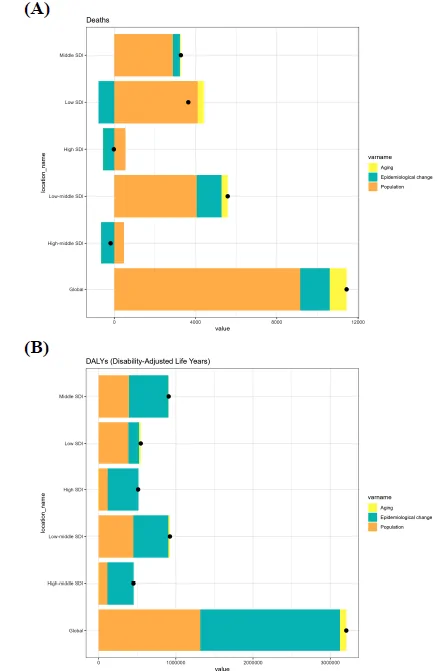
Decomposition analysis of dietary risk-attributable (**A)** deaths and (**B**) DALYs among women from 1990 to 2021, stratified by Socio-Demographic Index (SDI) regions. The figure illustrates the contributions of population growth, population aging, and age-specific rates to the total change over time. **Abbreviations:** DALYs, disability-adjusted life years; SDI, Socio-Demographic Index.

**Fig. (5):**
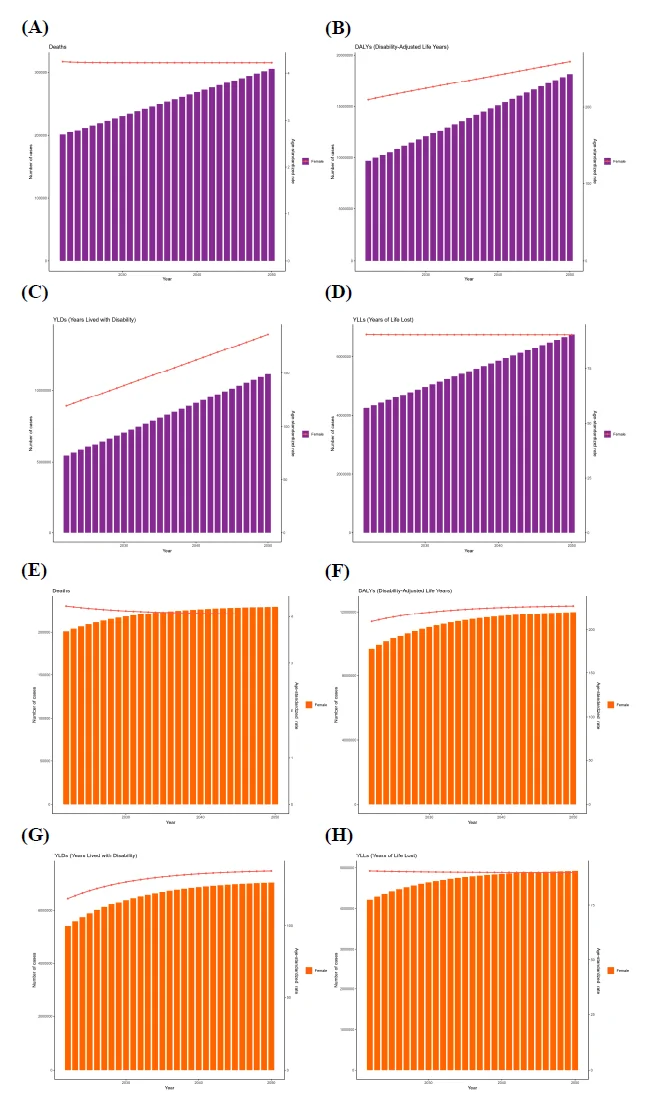
Forecasted trends in dietary risk-attributable deaths, DALYs, YLDs, and YLLs among women from 2022 to 2050, modeled using ARIMA and Exponential Smoothing (ES) approaches. Subfigures (**A**-**D**) present projections based on the ARIMA model (in purple), while E-H represent forecasts from the Exponential Smoothing model (in yellow). Within each model, indicators are shown in the following left-to-right, top-to-bottom order: (**A** & **E**) Deaths, (**B** & **F**) DALYs, (**C** & **G**) YLDs, and (**D** & **H**) YLLs. **Abbreviations:** DALYs, disability-adjusted life years; YLDs, years lived with disability; YLLs, years of life lost; ASMR, age-standardized mortality rate; ASDR, age-standardized DALY rate; ARIMA, autoregressive integrated moving average; ES, exponential smoothing.

**Table 1:** The number of deaths cases and the age-standardized deaths rates attributable to in 1990 and 2021, and its trends from 1990 to 2021 globally.

**Characteristics**	**1990**		**2021**		**1990-2021**
	**Number of deaths** **cases(95% UI)**	**The age-standardized deaths** **rate/100000(95% *UI*)**	**Number of deaths** **cases(95% *UI*)**	**The age-standardized deaths** **rate/100000(95% *UI*)**	**EAPC(95% CI)**
Global	90493 (16783-146848)	4.47 (0.83-7.26)	197699 (39636-324534)	4.25 (0.85-6.98)	-0.3 (-0.38--0.23)
**Age**					
25-29 years	376 (81-616)	0.17 (0.04-0.28)	532 (123-860)	0.18 (0.04-0.3)	0.07 (-0.03-0.17)
30-34 years	531 (110-841)	0.28 (0.06-0.44)	836 (204-1353)	0.28 (0.07-0.45)	-0.07 (-0.17-0.02)
35-39 years	779 (152-1270)	0.45 (0.09-0.73)	1354 (307-2175)	0.49 (0.11-0.78)	0.11 (-0.06-0.28)
40-44 years	1195 (219-1939)	0.85 (0.16-1.38)	2476 (519-3988)	1 (0.21-1.61)	0.32 (0.15-0.5)
45-49 years	2484 (477-4023)	2.18 (0.42-3.54)	5256 (1189-8506)	2.23 (0.5-3.61)	-0.13 (-0.24--0.01)
**SDI Regions**					
High-middle SDI	21335 (4564-33605)	3.96 (0.86-6.25)	39668 (8418-63093)	3.47 (0.74-5.51)	-0.6 (-0.75--0.44)
High SDI	28267 (5796-44741)	4.13 (0.85-6.53)	33043 (7316-52395)	2.46 (0.56-3.86)	-2.07 (-2.32--1.82)
Low-middle SDI	13688 (2341-22720)	5.25 (0.86-8.76)	46687 (9331-78071)	6.88 (1.35-11.55)	0.92 (0.87-0.96)
Low SDI	7859 (1037-13166)	8.01 (1.06-13.48)	18450 (2905-30965)	8.26 (1.28-14.01)	-0.02 (-0.09-0.05)
Middle SDI	19188 (3103-32850)	4.04 (0.66-6.92)	59594 (11187-100488)	4.36 (0.81-7.36)	0.24 (0.19-0.29)
**GBD Regions**					
Advanced Health System	43310 (9399-67468)	4.28 (0.93-6.65)	58211 (13388-90857)	3.13 (0.73-4.86)	-0.6 (-1.48-0.28)
Africa	9776 (1459-16302)	7.73 (1.16-12.85)	26970 (4910-44541)	9.34 (1.67-15.36)	0.8 (-0.47-2.08)
African Region	8624 (1197-14452)	8.54 (1.2-14.27)	22190 (3741-37003)	9.67 (1.62-16.09)	0.44 (-0.88-1.76)
America	21799 (4288-34876)	6.35 (1.25-10.17)	40443 (8520-65853)	5.36 (1.14-8.7)	-0.27 (-0.56-0.01)
Andean Latin America	440 (82-750)	4.38 (0.8-7.47)	1393 (255-2457)	4.55 (0.83-8.04)	1.06 (0.49-1.63)
Asia	29588 (4944-50176)	3.18 (0.54-5.44)	90296 (17795-152465)	3.47 (0.68-5.87)	1.28 (0.89-1.68)
Australasia	482 (90-777)	3.55 (0.67-5.71)	824 (159-1314)	2.4 (0.46-3.81)	-0.69 (-1.5-0.12)
Basic Health System	25622 (3891-43806)	3.7 (0.57-6.35)	70967 (12603-121045)	3.67 (0.65-6.25)	1.15 (0.77-1.53)
Caribbean	1448 (273-2388)	11.22 (2.11-18.52)	2373 (399-4095)	8.09 (1.36-13.99)	-0.36 (-0.6--0.12)
Central Africa	1219 (89-2217)	9.46 (0.68-16.85)	3378 (278-6004)	11.39 (0.91-20.56)	0.37 (-0.98-1.75)
Central Asia	986 (224-1473)	3.55 (0.81-5.3)	2993 (702-4650)	6.56 (1.55-10.16)	1.85 (1.49-2.22)
Central Europe	3779 (846-5868)	4.32 (0.97-6.69)	5899 (1356-9244)	4.05 (0.94-6.32)	0.95 (0.09-1.81)
Central Latin America	4241 (824-7051)	10.47 (2-17.46)	12446 (2846-20946)	9.28 (2.11-15.63)	0.64 (0.02-1.26)
Central Sub-Saharan Africa	1024 (50-1909)	10.13 (0.43-18.96)	2738 (160-5151)	10.87 (0.54-20.54)	0.06 (-1.29-1.43)
Commonwealth High Income	3404 (711-5386)	3.61 (0.75-5.68)	3416 (751-5390)	1.97 (0.44-3.09)	-1.57 (-2.48--0.64)
Commonwealth Low Income	2721 (217-4635)	8.09 (0.6-13.95)	8099 (1276-14177)	8.87 (1.33-15.52)	0.7 (-0.72-2.14)
Commonwealth Middle Income	13302 (2804-22125)	5.14 (1.09-8.61)	49922 (11206-82431)	6.79 (1.51-11.19)	1.68 (0.84-2.51)
East Asia	7838 (848-14046)	1.92 (0.22-3.43)	20373 (2284-37670)	1.78 (0.2-3.32)	0.99 (0.6-1.38)
East Asia & Pacific - WB	15675 (1946-27477)	2.42 (0.3-4.22)	36556 (5154-65273)	2.07 (0.29-3.68)	0.63 (0.32-0.95)
Eastern Africa	3068 (349-5189)	9.79 (1.08-16.88)	5261 (837-9036)	7.48 (1.14-12.87)	-1.19 (-2.62-0.26)
Eastern Europe	3179 (671-4826)	1.7 (0.36-2.57)	11430 (2620-17545)	4.73 (1.09-7.26)	2.58 (1.31-3.88)
Eastern Mediterranean Region	5368 (1210-8680)	7.32 (1.68-11.85)	20079 (4656-31985)	10.81 (2.52-17.29)	1.63 (0.51-2.78)
Eastern Sub-Saharan Africa	3493 (375-5922)	10.46 (1.07-18.17)	5843 (831-10096)	7.81 (1.05-13.51)	-1.33 (-2.79-0.16)
Europe	28907 (6389-44842)	4.39 (0.97-6.81)	39380 (9181-61079)	3.62 (0.85-5.61)	-0.07 (-1.02-0.88)
Europe & Central Asia - WB	29443 (6502-45657)	4.37 (0.97-6.77)	41396 (9623-64041)	3.77 (0.88-5.85)	0.01 (-0.87-0.89)
European Region	29734 (6582-46082)	4.39 (0.97-6.79)	42117 (9843-65081)	3.8 (0.89-5.89)	0.02 (-0.85-0.9)
High-income Asia Pacific	2206 (487-3534)	1.94 (0.43-3.11)	2325 (514-3897)	0.67 (0.14-1.11)	-1.78 (-2.9--0.64)
High-income North America	10584 (2272-16575)	4.95 (1.07-7.72)	13068 (3161-20148)	3.44 (0.84-5.25)	-1.44 (-2.23--0.64)
Latin America & Caribbean - WB	11474 (2051-18817)	8.61 (1.54-14.15)	27712 (5394-46002)	7.17 (1.4-11.91)	0.34 (-0.05-0.73)
Limited Health System	18862 (3161-31516)	5.72 (0.94-9.56)	61666 (12793-102600)	6.77 (1.4-11.26)	1.12 (0.18-2.07)
Middle East & North Africa - WB	2559 (638-4076)	5.05 (1.27-8.02)	10151 (2598-16229)	7.2 (1.85-11.52)	2.13 (1.1-3.16)
Minimal Health System	2543 (252-4356)	9.53 (0.95-16.42)	6599 (778-11357)	11.73 (1.35-20.3)	0.4 (-0.99-1.81)
North Africa and Middle East	4568 (1208-7364)	6.46 (1.72-10.37)	14579 (3696-23372)	7.53 (1.92-12.04)	1.68 (0.76-2.61)
North America	10586 (2273-16577)	4.95 (1.07-7.72)	13070 (3161-20151)	3.44 (0.84-5.25)	-1.44 (-2.23--0.64)
Northern Africa	1064 (263-1747)	4.18 (1.06-6.78)	4765 (1210-7911)	7.46 (1.88-12.36)	3.25 (2.27-4.25)
Oceania	271 (42-470)	20.83 (3.14-36.45)	798 (128-1370)	24.13 (3.92-41.12)	0.77 (-0.35-1.91)
Region of the Americas	21799 (4288-34876)	6.35 (1.25-10.17)	40443 (8520-65853)	5.36 (1.14-8.7)	-0.27 (-0.56-0.01)
South-East Asia Region	12054 (1949-20552)	4.05 (0.63-6.98)	42353 (8994-71518)	4.97 (1.04-8.41)	1.67 (0.93-2.41)
South Asia	11122 (2086-18796)	4.93 (0.88-8.33)	44159 (9643-73267)	6.55 (1.41-10.9)	1.8 (0.94-2.67)
South Asia - WB	11764 (2159-19826)	5.03 (0.89-8.48)	46045 (9949-76955)	6.63 (1.42-11.13)	1.76 (0.91-2.63)
Southeast Asia	5171 (609-9301)	4.15 (0.48-7.39)	13353 (2338-23362)	4.07 (0.71-7.1)	0.88 (0.32-1.44)
Southern Africa	2092 (263-3578)	9.64 (1.18-16.51)	7188 (1176-12147)	15.04 (2.42-25.4)	2.06 (1.01-3.12)
Southern Latin America	2073 (467-3141)	8.1 (1.83-12.27)	2620 (634-3976)	4.96 (1.21-7.5)	-1.02 (-1.39--0.66)
Southern Sub-Saharan Africa	1439 (202-2489)	9.96 (1.41-17.26)	5845 (993-9821)	18.8 (3.16-31.49)	3.26 (2.52-4.01)
Sub-Saharan Africa - WB	8765 (1186-14693)	8.68 (1.18-14.57)	22317 (3661-37388)	9.89 (1.61-16.5)	0.4 (-0.93-1.76)
Tropical Latin America	3324 (452-5712)	7.35 (0.98-12.7)	8964 (1249-15225)	6.29 (0.87-10.69)	0.72 (0.31-1.12)
Western Africa	2332 (544-3876)	6.73 (1.58-11.21)	6378 (1454-10709)	8.23 (1.85-13.82)	0.46 (-0.94-1.88)
Western Europe	20189 (4352-31386)	5.23 (1.13-8.1)	18347 (4182-28863)	2.52 (0.57-3.93)	-1.56 (-2.79--0.32)
Western Pacific Region	11956 (1565-20919)	2.17 (0.29-3.79)	28719 (3736-51735)	1.87 (0.24-3.37)	0.82 (0.47-1.17)
Western Sub-Saharan Africa	2636 (608-4352)	6.86 (1.6-11.38)	7329 (1632-12443)	8.49 (1.88-14.33)	0.48 (-0.92-1.9)
World Bank High Income	38014 (8083-59349)	4.74 (1.01-7.38)	42574 (9574-66810)	2.71 (0.62-4.23)	-1.28 (-2.23--0.32)
World Bank Low Income	5406 (608-9202)	7.9 (0.87-13.58)	11230 (1512-19196)	7.33 (0.96-12.55)	-0.43 (-1.67-0.83)
World Bank Lower Middle Income	21597 (3959-36038)	4.66 (0.83-7.77)	74450 (15685-124477)	6.33 (1.32-10.61)	1.65 (0.87-2.44)
World Bank Upper Middle Income	25318 (4343-42265)	3.39 (0.59-5.68)	69186 (12991-116502)	3.67 (0.69-6.18)	1.24 (0.96-1.53)

**Table 2:** The number of DALYs cases and the age-standardized DALYs rates attributable to in 1990 and 2021, and its trends from 1990 to 2021 globally.

**Characteristics**	**1990**		**2021**		**1990-2021**
	**Number of DALYs** **cases(95% *UI*)**	**The age-standardized DALYs** **rate/100000(95% *UI*)**	**Number of DALYs** **cases(95% *UI*)**	**The age-standardized DALYs** **rate/100000(95% *UI*)**	**EAPC(95% CI)**
Global	3315616 (640715-5432411)	154.61 (29.91-253.1)	9396286 (2069187-15612534)	207.18 (45.75-344.06)	1.44 (1.27-1.61)
**Age**					
25-29 years	63669 (14082-110537)	28.93 (6.4-50.22)	164853 (43516-290062)	56.65 (14.95-99.68)	2.27 (2.19-2.35)
30-34 years	81205 (16615-139122)	42.71 (8.74-73.18)	245862 (60284-428279)	82.25 (20.17-143.27)	2.12 (2.06-2.19)
35-39 years	109931 (22719-186130)	63.38 (13.1-107.31)	326077 (77774-553780)	117.38 (28-199.34)	1.99 (1.93-2.04)
40-44 years	137151 (26535-232636)	97.81 (18.92-165.9)	434300 (102590-743738)	175.06 (41.35-299.79)	1.85 (1.77-1.94)
45-49 years	204409 (41242-336807)	179.62 (36.24-295.96)	644611 (156014-1084578)	273.55 (66.21-460.26)	1.25 (1.17-1.33)
**SDI Regions**					
High-middle SDI	821678 (182667-1345461)	146.79 (32.73-240.25)	1906415 (425718-3182384)	184.15 (41.21-308.12)	0.58 (0.5-0.67)
High SDI	910006 (201728-1464326)	147.34 (32.94-236.58)	2025651 (503351-3353452)	206.16 (52.39-338.37)	0.92 (0.82-1.02)
Low-middle SDI	507909 (90094-850902)	162.62 (28.46-273.91)	1911320 (434741-3218006)	246.65 (55.18-418.24)	1.39 (1.33-1.45)
Low SDI	278146 (40088-461913)	236.8 (33.54-395.21)	777284 (135466-1294966)	276.75 (47.46-467.42)	0.37 (0.31-0.43)
Middle SDI	792048 (133716-1362862)	142.73 (24.01-246.68)	2764100 (579049-4707427)	192.93 (40.41-328.66)	0.92 (0.89-0.95)
**GBD Regions**					
Advanced Health System	1446987 (338715-2301632)	154.57 (36.5-245.26)	3028341 (778495-4930835)	215.24 (56.47-346.25)	1.33 (0.69-1.97)
Africa	337023 (55414-556481)	226.11 (36.75-374.82)	1115950 (231684-1872312)	308.79 (61.78-516.82)	1.25 (0.23-2.27)
African Region	288817 (43615-477770)	244.5 (36.41-405.57)	856953 (159539-1430127)	299.05 (53.83-503.33)	0.77 (-0.3-1.85)
America	772063 (163691-1258725)	231.98 (49.19-378.05)	2138017 (511263-3536506)	306.11 (73.83-506.45)	1.3 (1.1-1.5)
Andean Latin America	15595 (3218-27136)	142.45 (28.49-249.08)	58167 (12045-101297)	185.47 (37.83-324.02)	1.67 (1.19-2.15)
Asia	1268493 (219338-2183435)	118.03 (20.38-202.73)	4484326 (895405-7597905)	169.46 (33.86-287.04)	1.98 (1.71-2.26)
Australasia	15720 (2974-25533)	123.3 (23.72-200.47)	37712 (7715-63376)	141.99 (29.67-235.96)	0.84 (0.29-1.39)
Basic Health System	1084881 (176903-1867663)	135.93 (21.94-233.66)	3610674 (697374-6234293)	182.78 (35.45-315)	1.91 (1.64-2.18)
Caribbean	49518 (10000-82706)	363.56 (72.63-608.05)	105235 (18520-182949)	375.56 (66.23-651.77)	0.69 (0.48-0.89)
Central Africa	41355 (3374-73312)	263.03 (20.36-465.46)	133215 (12659-233142)	351.54 (29.94-616.21)	0.79 (-0.3-1.89)
Central Asia	47524 (11833-75043)	169.17 (42.11-267.61)	171607 (44454-273711)	352.1 (91.28-564.02)	2.57 (2.29-2.85)
Central Europe	165084 (40047-265900)	193.08 (47.12-309.41)	280657 (68453-463273)	234.11 (58.18-383.85)	1.47 (0.76-2.18)
Central Latin America	166535 (35787-279498)	358.82 (74.48-604.78)	546421 (133891-932181)	395.65 (96.39-674.99)	1.17 (0.69-1.65)
Central Sub-Saharan Africa	35794 (2012-65608)	283.22 (14.19-521.95)	109948 (8400-194576)	338.49 (21.6-599.95)	0.47 (-0.6-1.55)
Commonwealth High Income	104159 (23581-166521)	125.96 (28.83-200.9)	220663 (54448-367937)	177.49 (44.91-293.41)	1.11 (0.5-1.73)
Commonwealth Low Income	90089 (8097-154831)	210.65 (17.19-361.94)	295385 (49932-503874)	247.86 (40.68-427.77)	1.06 (0-2.14)
Commonwealth Middle Income	490334 (104511-819694)	156.74 (32.88-262.58)	1952622 (482156-3225833)	233.02 (56.81-385.45)	1.94 (1.33-2.55)
East Asia	411408 (49809-747972)	87.74 (10.65-159.45)	1332771 (166728-2437923)	123.46 (15.97-224.18)	2.09 (1.78-2.4)
East Asia & Pacific - WB	724456 (98383-1288098)	99.46 (13.51-175.65)	2191156 (342370-3902889)	130.9 (20.77-234.93)	1.8 (1.53-2.06)
Eastern Africa	102960 (12512-173104)	277.63 (32.66-469.44)	202081 (37191-337828)	226.49 (39.89-383.39)	-0.86 (-2.03-0.34)
Eastern Europe	188553 (42929-299377)	107.47 (24.48-169.7)	439098 (104624-695448)	207.73 (50.08-328.49)	1.93 (1.18-2.68)
Eastern Mediterranean Region	206909 (48901-333663)	234.67 (54.84-378.73)	1005718 (266158-1649559)	424.83 (111.1-693.88)	2.44 (1.59-3.31)
Eastern Sub-Saharan Africa	114081 (12998-192278)	285.22 (31.07-485.57)	212160 (34221-357806)	222.52 (33.72-379.08)	-1.08 (-2.28-0.14)
Europe	923774 (220103-1463650)	151.64 (36.51-239.91)	1633118 (420030-2631700)	198.56 (52.02-317.59)	1.14 (0.43-1.84)
Europe & Central Asia - WB	952651 (227036-1512176)	152.26 (36.65-241.13)	1754870 (449688-2823082)	206.77 (53.83-330.07)	1.23 (0.58-1.88)
European Region	961973 (229874-1526602)	152.81 (36.88-241.94)	1779469 (457431-2860955)	207.47 (54.16-331.04)	1.23 (0.58-1.87)
High-income Asia Pacific	115960 (28605-193961)	103.07 (25.5-172.34)	294342 (68919-525243)	153.96 (36.85-268.54)	2.05 (1.3-2.79)
High-income North America	357319 (81432-566208)	187.99 (43.31-296.58)	933756 (249208-1515771)	291.26 (78.93-472.96)	1.52 (0.97-2.08)
Latin America & Caribbean - WB	422881 (82975-707384)	288.94 (55.67-484.79)	1216832 (264435-2060413)	317.04 (68.92-536.52)	1.07 (0.75-1.39)
Limited Health System	687604 (124029-1147142)	171.72 (30.61-286.08)	2461187 (568961-4111091)	231.16 (52.49-387.29)	1.49 (0.79-2.2)
Middle East & North Africa - WB	108446 (29014-174327)	175.38 (46.87-284.17)	593561 (175692-985577)	333.03 (97.56-555.89)	3.02 (2.28-3.77)
Minimal Health System	90315 (9871-153916)	281.38 (30.05-481.79)	284568 (37736-493545)	392.42 (49.05-683.02)	0.9 (-0.22-2.03)
North Africa and Middle East	174453 (47863-283062)	205.9 (56.59-335.46)	815944 (240016-1350401)	343.08 (99.7-567.95)	2.63 (1.93-3.33)
North America	357364 (81436-566296)	188 (43.31-296.6)	933833 (249210-1515903)	291.26 (78.93-472.96)	1.52 (0.97-2.08)
Northern Africa	47383 (12455-78038)	149.78 (39.42-248.16)	276410 (77990-465010)	345.08 (96.66-578.58)	3.78 (3.1-4.46)
Oceania	9946 (1556-17095)	620.37 (95.14-1076.59)	33339 (6109-56101)	800.83 (144.38-1347.13)	1.17 (0.32-2.02)
Region of the Americas	772063 (163691-1258725)	231.98 (49.19-378.05)	2138017 (511263-3536506)	306.11 (73.83-506.45)	1.3 (1.1-1.5)
South-East Asia Region	463297 (78466-805014)	127.55 (21.42-221.77)	1680599 (399462-2803068)	174.97 (41.04-292.61)	1.83 (1.31-2.35)
South Asia	424428 (79155-715898)	149.76 (27.61-253.84)	1747009 (425561-2877575)	225.1 (53.96-371.33)	2.06 (1.46-2.67)
South Asia - WB	449873 (82891-758778)	154 (28.19-260.34)	1843106 (444148-3050030)	230.99 (54.83-383.65)	2.05 (1.45-2.65)
Southeast Asia	182071 (23356-323163)	128.77 (16.06-228.5)	538132 (103282-949511)	149.87 (28.74-265.63)	1.32 (0.89-1.75)
Southern Africa	67076 (9282-111901)	272.36 (36.33-456.43)	238453 (40327-409275)	431.94 (71.58-740.05)	2.12 (1.25-2.99)
Southern Latin America	66313 (16770-101962)	258.09 (65.44-397.2)	133701 (35584-217527)	284.2 (76.38-462.33)	0.62 (0.38-0.86)
Southern Sub-Saharan Africa	45307 (6393-76659)	287.84 (39.9-489.77)	185385 (31503-316498)	544.08 (92.11-932.11)	3.28 (2.67-3.89)
Sub-Saharan Africa - WB	291283 (42714-482981)	247.33 (35.75-410.63)	843394 (151477-1416055)	299.35 (52.83-503.41)	0.69 (-0.41-1.8)
Tropical Latin America	126675 (18609-215938)	248.79 (35.81-426.64)	377012 (59058-646813)	266.2 (41.73-456.39)	1.2 (0.89-1.51)
Western Africa	78249 (17750-127684)	195.76 (44.65-319.62)	265791 (63484-444332)	263.77 (61.36-447.03)	0.97 (-0.17-2.11)
Western Europe	514913 (119774-812910)	153.63 (36.28-242.88)	739511 (186950-1201550)	167.59 (43.71-270.62)	0.44 (-0.4-1.29)
Western Pacific Region	590591 (87068-1053888)	95.01 (14.04-168.69)	1861903 (275476-3367472)	132.74 (20.06-240.59)	2.05 (1.76-2.34)
Western Sub-Saharan Africa	88419 (19651-144062)	199.21 (44.53-324.8)	304377 (71231-509861)	271.54 (61.83-459.51)	0.98 (-0.16-2.13)
World Bank High Income	1190261 (273502-1899294)	164.45 (38.13-261.78)	2406115 (610389-3947250)	215.61 (56.05-349.43)	1.16 (0.5-1.83)
World Bank Low Income	190725 (23330-322300)	236.5 (28.33-400.72)	486409 (76940-829503)	258.34 (39.27-444.58)	0.18 (-0.83-1.19)
World Bank Lower Middle Income	820983 (151907-1375373)	151.24 (27.74-252.98)	3121416 (741478-5216186)	230.47 (53.95-386.89)	1.97 (1.39-2.55)
World Bank Upper Middle Income	1107748 (202904-1885023)	134.22 (24.55-227.85)	3370701 (659838-5799041)	183.77 (36.24-316.38)	1.81 (1.57-2.05)

## Data Availability

The datasets analysed during the current study are available in the [Global Burden of Disease Study] repository, [https://ghdx.healthdata.org/gbd-2021/sources]

## LIST OF ABBREVIATIONS

T2D: Type 2 Diabetes
DALYs: Disability-adjusted Life Years
GBD: Global Burden of Disease
SDI: Socio-demographic Index
