# Supplementary material for: The Global Burden of Type 2 Diabetes Attributable to Dietary Risks Among Women: A 1990-2021 Analysis and Forecasting Through 2050
Source: Curr Diabetes Rev. 2026 Feb 25;22(7):E15733998409251. doi: 10.2174/0115733998409251251105102446 (PMC13717586; doi:10.2174/0115733998409251251105102446)
Supplement: Supplementary file 1 [file CDR-22-7-E15733998409251_SD1.pdf]

Supplementary Material

The Global Burden of Type 2 Diabetes Attributable to Dietary Risks Among Women: A 1990-2021 Analysis and Forecasting Through 2050

Tao Ding[1,2], Xiaoling Wang[1,2], Puwen Chen[2], Mengqi Zhong[2], Fuzhen Liu[3] and Yu Xia[1,2,*]

[1]Laboratory Medicine, First Affiliated Hospital of Gannan Medical University, Ganzhou, 34100, China; [2]The First School of Clinical Medicine, Gannan Medical University, Ganzhou, 34100, China; [3]School of Medical Technology, Gannan Medical University, Ganzhou, China

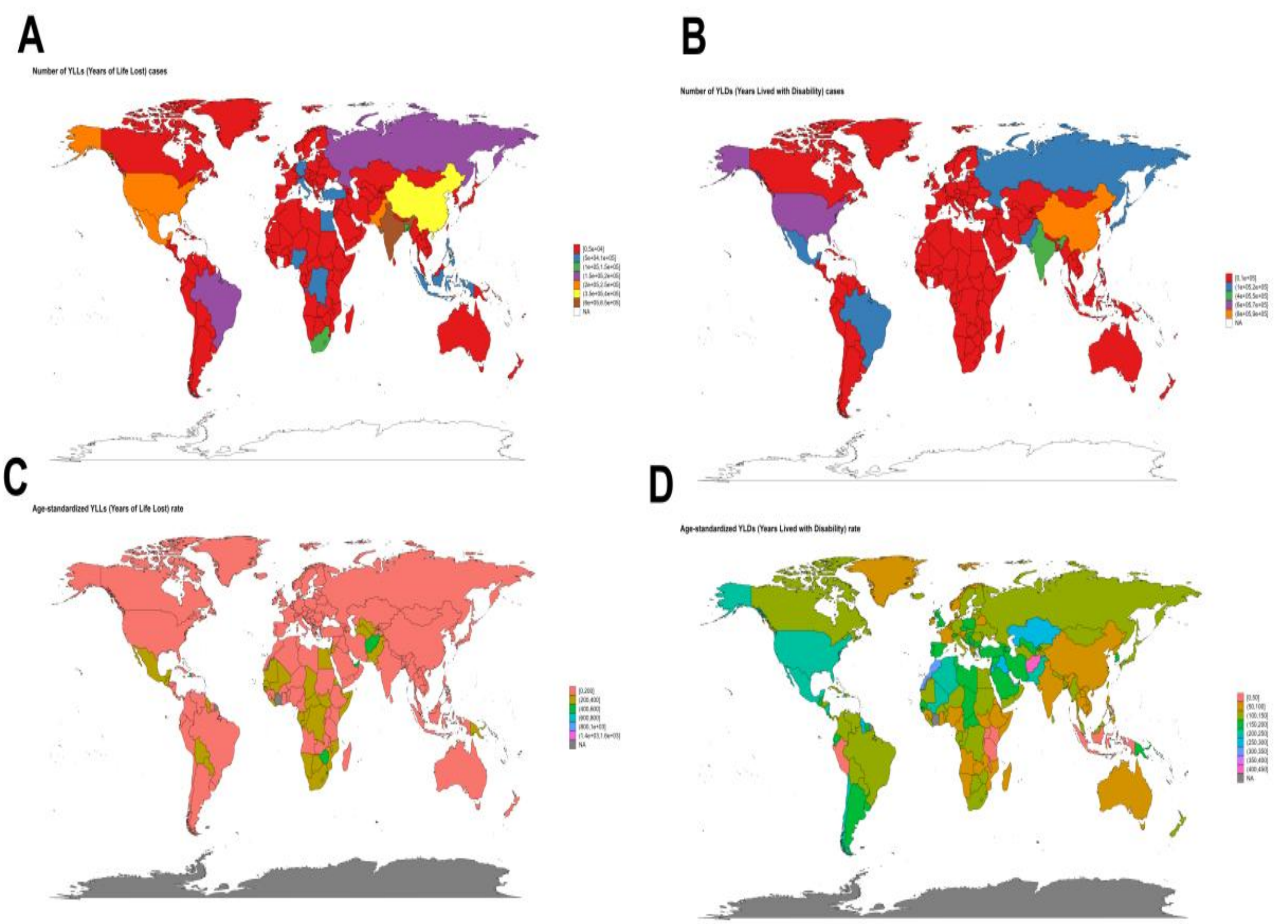

Fig. (S1). Numbers and age-standardized rates of dietary risk-attributable years of life lost (YLLs) and years lived with disability (YLDs) among women across countries and territories in 2021.A & B: Number of YLLs and YLDs.C & D: Age-standardized rates of YLLs and YLDs.Abbreviations: YLLs, years of life lost; YLDs, years lived with disability.

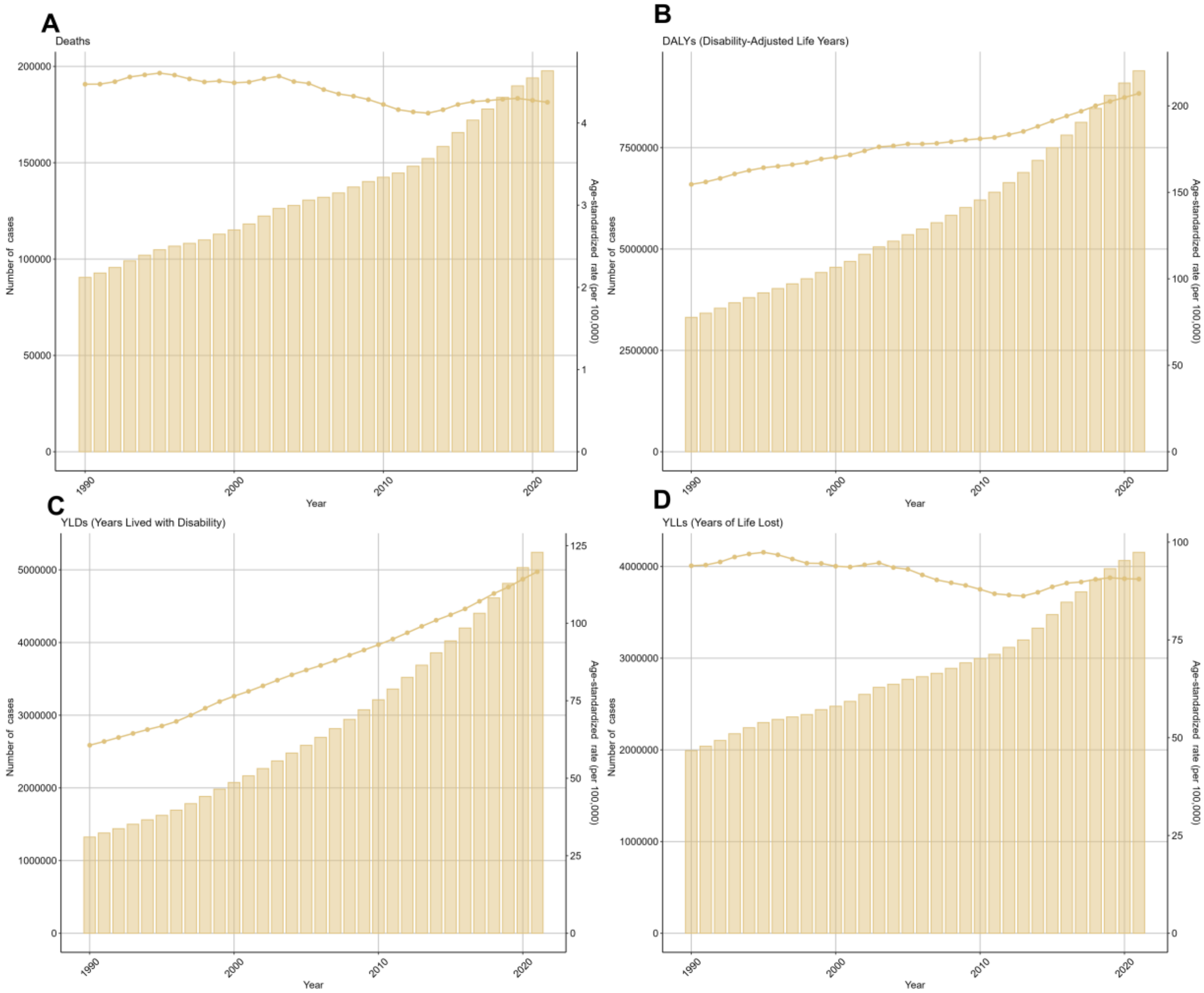

Fig. (S2). Trends in dietary risk-attributable deaths, DALYs, YLDs, and YLLs among women globally from 1990 to 2021. Subfigures A－D respectively represent deaths (A), disability-adjusted life years (B), years lived with disability (C), and years of life lost (D). Each panel presents both the absolute number of cases (bars) and age-standardized rates (lines). **Abbreviations:** DALYs, disability-adjusted life years; YLLs, years of life lost; YLDs, years lived with disability.

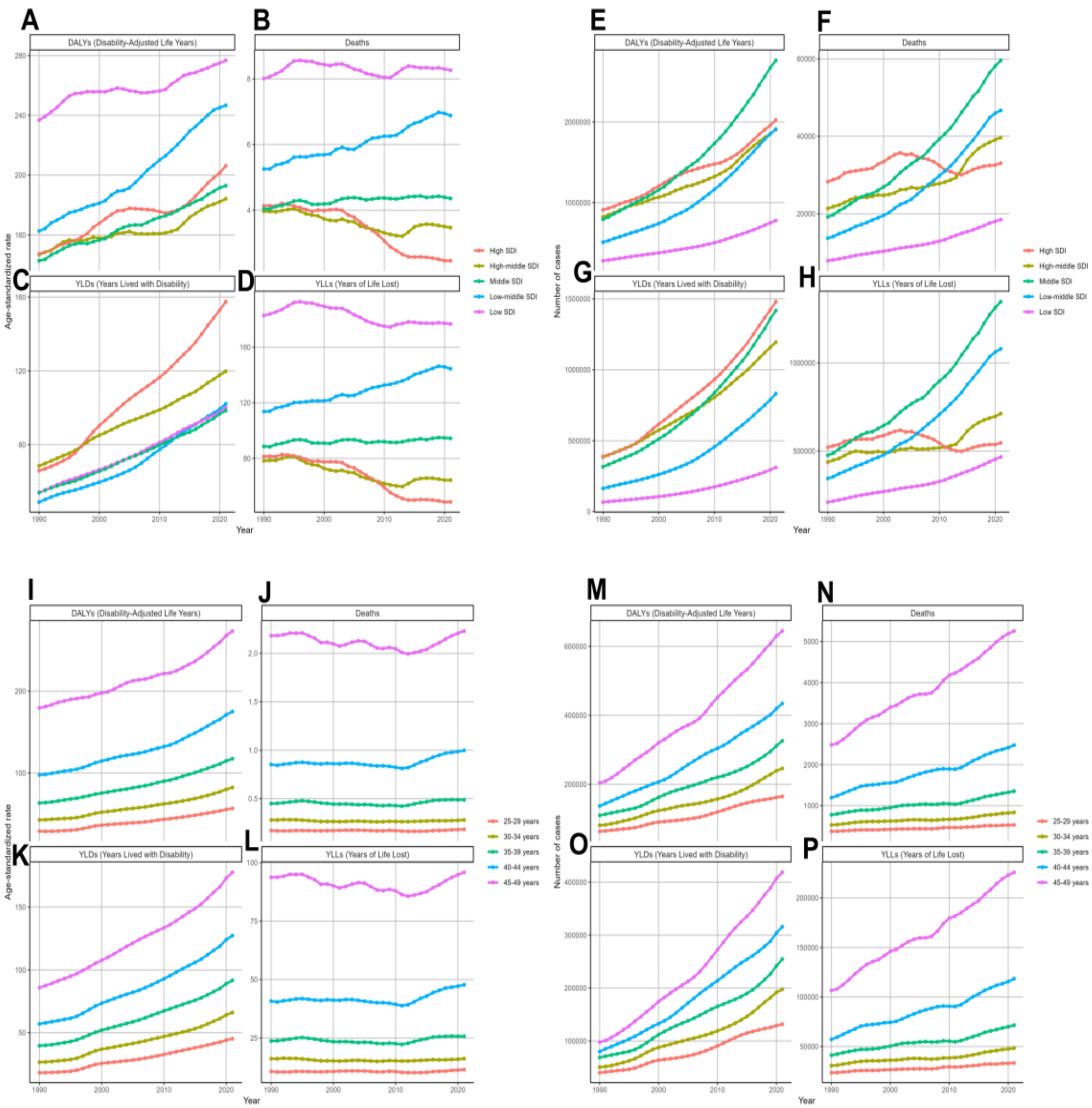

Fig. (S3). Trends in dietary risk-attributable DALYs, deaths, YLDs, and YLLs among women from 1990 to 2021, stratified by SDI region (A – H) and age group (I – P). Subfigures A – D and I – L present age-standardized rates, while E – H and M – P present the number of cases. **Abbreviations**: DALYs, disability-adjusted life years; YLLs, years of life lost; YLDs, years lived with disability; SDI, Socio-Demographic Index.

Table S1 The number of deaths cases and the age-standardized deaths rates attributable to in 1990 and 2021, and its trends from 1990 to 2021 across countries and territories.

Characteristi-cs	1990		2021		1990–2021
	Number of deaths cases(95% UI)	The age-standardized deaths rate/100000(95% UI)	Number of deaths cases(95% UI)	The age-standardized deaths rate/100000(95% UI)	EAPC(95% CI)
Afghanistan	398 (45-722)	12.66 (1.45-22.85)	969 (134-1739)	20.77 (2.9-37)	0.54 (-0.73-1.82)
Albania	19 (6-30)	1.93 (0.57-2.98)	47 (14-74)	2.01 (0.58-3.15)	2.3 (1.79-2.81)
Algeria	157 (37-268)	3.81 (0.96-6.66)	766 (203-1283)	5.85 (1.53-9.63)	3 (1.91-4.1)
American Samoa	2 (0-3)	16.77 (2.87-29)	6 (1-10)	26.37 (5.23-46.74)	2.72 (1.9-3.55)
Andorra	1 (0-1)	3.34 (0.85-5.52)	2 (1-4)	2.54 (0.61-4.27)	0.78 (0.05-1.52)
Angola	162 (14-282)	9.7 (0.79-17.01)	345 (63-617)	6.42 (1.17-11.54)	-2.18 (-3.73--0.6)
Antigua and Barbuda	5 (1-8)	14.84 (3.45-24.34)	7 (2-12)	13.6 (3.16-21.57)	-0.67 (-0.89--0.44)
Argentina	1567 (342-2393)	8.71 (1.91-13.31)	1779 (425-2714)	5.3 (1.28-8.04)	-1.31 (-1.67--0.95)
Armenia	96 (23-151)	6.29 (1.53-9.85)	125 (28-195)	4.89 (1.08-7.68)	-0.08 (-0.96-0.8)
Australia	412 (77-663)	3.63 (0.69-5.8)	720 (139-1143)	2.45 (0.47-3.88)	-0.58 (-1.42-0.26)
Austria	375 (62-611)	4.45 (0.71-7.23)	347 (71-563)	2.52 (0.51-4.08)	-0.45 (-1.73-0.85)
Azerbaijan	109 (29-172)	3.75 (1.01-5.89)	362 (96-604)	6.56 (1.76-10.98)	1.9 (1.5-2.3)
Bahamas	11 (2-18)	12.51 (2.01-20.89)	18 (3-30)	8.41 (1.67-14.24)	-0.96 (-1.37--0.55)
Bahrain	14 (4-23)	24.1 (6.57-38.78)	76 (19-125)	30.87 (7.55-50.37)	0.95 (-0.69-2.62)
Bangladesh	1513 (95-2633)	9.08 (0.53-15.9)	5493 (871-9658)	10.62 (1.52-18.77)	1.53 (0.11-2.96)
Barbados	38 (7-62)	21.11 (3.75-34.76)	42 (8-70)	14.17 (2.83-23.91)	-0.91 (-1.54--0.28)
Belarus	155 (32-238)	1.82 (0.38-2.79)	154 (31-243)	1.43 (0.29-2.26)	-2.31 (-3.36--1.25)
Belgium	456 (102-714)	4.44 (1-6.96)	294 (69-463)	1.64 (0.39-2.57)	-2.52 (-3.74--1.28)

Belize	4 (1-8)	9.55 (1.94-16.6)	13 (3-22)	9.18 (2.23-15.53)	0.15 (-0.76-1.07)
Benin	50 (12-86)	5.38 (1.23-9.24)	170 (38-295)	7.1 (1.54-12.39)	0.76 (-0.65-2.18)
Bermuda	2 (0-4)	7.02 (0.85-12.22)	3 (0-5)	3.2 (0.38-5.76)	-1.55 (-2.3--0.79)
Bhutan	6 (1-10)	5.41 (0.86-9.75)	19 (4-33)	6.67 (1.3-11.79)	1.83 (0.9-2.76)
Bolivia (Plurinational State of)	166 (26-287)	10.34 (1.6-17.82)	438 (63-819)	9.7 (1.47-18.14)	0.68 (-0.02-1.39)
Bosnia and Herzegovina	99 (25-159)	4.66 (1.17-7.46)	310 (92-522)	8.12 (2.42-13.55)	3.72 (2.95-4.49)
Botswana	38 (3-73)	14.32 (1.18-26.55)	96 (11-173)	13.44 (1.55-24.29)	1.37 (0.25-2.5)
Brazil	3253 (450-5587)	7.38 (1.01-12.73)	8612 (1228-14695)	6.17 (0.88-10.54)	0.65 (0.25-1.05)
Brunei Darussalam	8 (1-13)	18.17 (3.12-30.65)	16 (3-27)	11.07 (2.45-18.81)	-0.45 (-1.64-0.75)
Bulgaria	450 (109-679)	6.91 (1.7-10.43)	506 (111-794)	5.62 (1.29-8.72)	-0.17 (-1.16-0.83)
Burkina Faso	167 (24-287)	9.04 (1.27-15.34)	343 (53-583)	8.2 (1.26-14.1)	-0.43 (-1.78-0.94)
Burundi	112 (11-203)	9.89 (0.96-18.3)	171 (26-323)	9 (1.26-16.84)	-1.61 (-3.12--0.08)
Cabo Verde	3 (1-5)	2.09 (0.37-3.57)	17 (3-29)	6.5 (1.23-11.39)	3.67 (3.15-4.19)
Cambodia	118 (7-222)	4.92 (0.25-9.35)	380 (30-745)	5.75 (0.44-11.12)	1.25 (0.42-2.09)
Cameroon	117 (26-210)	5.91 (1.25-10.55)	431 (98-781)	7.73 (1.78-13.78)	0.71 (-0.71-2.15)
Canada	697 (121-1119)	3.61 (0.63-5.77)	894 (179-1413)	1.95 (0.41-3.08)	-1.81 (-2.77--0.84)
Central African Republic	77 (2-140)	14.63 (0.18-26.65)	163 (4-306)	16.27 (0.37-30.46)	0.2 (-1.21-1.64)
Chad	128 (18-223)	9.71 (1.35-16.69)	381 (65-636)	17.11 (2.97-28.38)	1.01 (-0.56-2.6)
Chile	344 (91-517)	6.47 (1.7-9.71)	612 (156-931)	4.01 (1.03-6.08)	-0.23 (-0.65-0.19)
China	7173 (776-12871)	1.83 (0.21-3.27)	18861 (2075-35030)	1.71 (0.19-3.19)	1.1 (0.72-1.48)

Colombia	338 (50-591)	3.96 (0.57-6.89)	758 (155-1323)	2.44 (0.5-4.27)	-0.9 (-1.42--0.37)
Comoros	9 (0-17)	9.79 (0.05-19.11)	23 (1-45)	9.94 (0.51-19.41)	0.75 (-0.27-1.79)
Congo	70 (3-129)	13.62 (0.47-25.69)	168 (13-311)	14.09 (0.83-25.41)	-0.05 (-1.24-1.16)
Cook Islands	2 (0-3)	32.52 (6.18-55.86)	3 (1-5)	22.97 (5.03-40.55)	0.24 (-0.16-0.63)
Costa Rica	38 (7-65)	4.38 (0.79-7.39)	127 (32-213)	4.18 (1.06-6.98)	0.16 (-0.61-0.94)
C么te d'Ivoire	84 (17-141)	5.85 (1.2-9.76)	396 (92-704)	9.09 (2.04-16.35)	2.4 (0.64-4.2)
Croatia	99 (28-161)	2.82 (0.78-4.56)	208 (51-341)	3.31 (0.84-5.4)	1.05 (-0.04-2.15)
Cuba	291 (66-468)	5.71 (1.3-9.22)	224 (58-377)	2.01 (0.52-3.38)	-2.17 (-2.89--1.44)
Cyprus	100 (27-157)	29.1 (7.8-46.85)	96 (22-160)	9.7 (2.28-16.04)	-3.36 (-3.67--3.04)
Czechia	349 (71-562)	4.02 (0.82-6.48)	721 (156-1203)	4.96 (1.08-8.26)	3.57 (2.37-4.78)
Democratic People's Republic of Korea	131 (19-242)	1.42 (0.21-2.62)	325 (52-609)	1.67 (0.27-3.16)	1.71 (1.4-2.02)
Democratic Republic of the Congo	675 (26-1302)	9.61 (0.27-18.62)	1990 (37-3822)	11.76 (0.14-22.71)	0.64 (-0.67-1.97)
Denmark	146 (27-237)	2.85 (0.52-4.6)	230 (51-363)	2.86 (0.65-4.47)	0.54 (-0.63-1.71)
Djibouti	5 (1-8)	9.13 (1.74-15.6)	25 (5-47)	11.15 (2.27-19.98)	1.21 (-0.36-2.81)
Dominica	8 (2-12)	20.69 (5.38-34.02)	10 (3-16)	21.63 (5.78-35.49)	0.27 (-0.02-0.57)
Dominican Republic	60 (14-105)	3.63 (0.83-6.33)	210 (46-373)	4.04 (0.88-7.21)	1.91 (1.31-2.52)
Ecuador	154 (30-264)	6.06 (1.14-10.41)	543 (91-934)	6.43 (1.08-11.05)	1.17 (0.5-1.85)
Egypt	545 (129-928)	5.55 (1.37-9.46)	2312 (542-4031)	12.04 (2.75-20.61)	3.8 (2.33-5.29)
El Salvador	63 (12-106)	4.04 (0.72-6.82)	268 (73-460)	7.32 (1.99-12.56)	2.99 (2.56-3.43)
Equatorial Guinea	11 (0-20)	11.25 (0.43-21.99)	23 (4-45)	9.35 (1.57-18.37)	-1.06 (-2.43-0.33)

Eritrea	61 (4-115)	11.54 (0.68-21.41)	158 (16-295)	12.3 (1.13-22.69)	0.8 (-0.66-2.28)
Estonia	21 (4-31)	1.51 (0.32-2.27)	62 (14-100)	3.17 (0.72-5.05)	2.15 (0.89-3.43)
Eswatini	25 (2-44)	17.44 (1.04-31.07)	73 (4-140)	24.65 (1.26-47.54)	2.38 (0.99-3.79)
Ethiopia	1629 (209-2739)	19.89 (2.51-33.86)	1673 (319-2836)	9.25 (1.67-15.75)	-3.16 (-4.74--1.55)
Fiji	84 (11-148)	49.39 (6.36-86.11)	252 (41-437)	66.68 (10.98-115.81)	1.87 (1.12-2.62)
Finland	113 (20-181)	2.28 (0.42-3.65)	95 (21-149)	1.04 (0.22-1.62)	-1.96 (-3.21--0.69)
France	2073 (407-3205)	3.55 (0.69-5.47)	2860 (619-4476)	2.34 (0.51-3.64)	-0.37 (-1.68-0.95)
Gabon	29 (0-56)	10.02 (-0.08-19.33)	49 (4-97)	10.21 (0.7-20.23)	-0.4 (-1.29-0.5)
Gambia	10 (1-18)	7.38 (1-12.96)	55 (9-97)	12.39 (1.95-21.88)	2.08 (0.6-3.58)
Georgia	133 (33-204)	3.4 (0.85-5.21)	219 (58-341)	5.74 (1.52-8.91)	3.57 (2.64-4.52)
Germany	4949 (1032-7965)	5.43 (1.12-8.69)	3942 (933-6277)	2.69 (0.63-4.21)	-1.78 (-3.11--0.42)
Ghana	179 (43-302)	6.41 (1.48-10.79)	639 (153-1123)	8.04 (1.93-14.02)	1.23 (0.04-2.44)
Greece	300 (72-461)	3.58 (0.86-5.47)	365 (87-568)	2.24 (0.54-3.44)	-0.44 (-1.52-0.65)
Greenland	1 (0-1)	5.17 (1.16-8.24)	1 (0-1)	2.42 (0.62-3.82)	-1.96 (-2.43--1.49)
Grenada	12 (2-20)	25.33 (4.83-41.95)	11 (2-18)	18.11 (4.06-30.22)	-0.43 (-0.68--0.17)
Guam	4 (1-6)	12.28 (2.94-20.63)	5 (1-9)	4.65 (1.27-7.67)	-1.08 (-1.62--0.54)
Guatemala	46 (11-75)	3.03 (0.71-4.95)	651 (171-1097)	11.19 (2.96-18.88)	4.48 (3.34-5.63)
Guinea	72 (14-125)	4.76 (0.96-8.53)	195 (41-359)	7.81 (1.65-14.6)	1.35 (0.24-2.47)
Guinea-Bissau	18 (1-32)	10.09 (0.35-18.23)	50 (3-90)	15.38 (0.88-28.77)	1.28 (-0.27-2.85)
Guyana	31 (5-53)	16.67 (2.56-29.06)	46 (12-82)	14.14 (3.7-24.91)	0.38 (-0.4-1.16)

Haiti	354 (43-630)	23.3 (2.69-41.85)	824 (58-1643)	24.95 (1.4-50.02)	0.42 (-0.62-1.47)
Honduras	25 (4-44)	2.46 (0.39-4.34)	170 (43-313)	5.27 (1.35-9.79)	3.18 (2.18-4.19)
Hungary	391 (86-621)	4.37 (0.96-6.92)	542 (148-839)	4.07 (1.1-6.3)	0.97 (-0.08-2.03)
Iceland	4 (1-6)	2.08 (0.48-3.18)	4 (1-6)	1.08 (0.22-1.68)	-1.48 (-2.21--0.75)
India	6914 (1435-11885)	3.61 (0.74-6.22)	28579 (6580-47650)	5.07 (1.16-8.48)	2.18 (1.41-2.95)
Indonesia	1473 (122-2732)	3.12 (0.25-5.8)	2802 (529-5096)	2.57 (0.47-4.7)	0 (-0.66-0.66)
Iran (Islamic Republic of)	380 (91-625)	3.94 (0.95-6.48)	1988 (546-3167)	5.85 (1.61-9.35)	3.24 (2.26-4.24)
Iraq	319 (84-506)	8.18 (2.16-13.06)	987 (253-1665)	9.56 (2.51-16.11)	0.63 (-0.46-1.73)
Ireland	86 (16-136)	3.62 (0.65-5.67)	65 (13-102)	1.32 (0.27-2.08)	-2.34 (-2.94--1.73)
Israel	193 (56-292)	7.7 (2.28-11.6)	491 (144-751)	6.08 (1.78-9.31)	-0.49 (-1.19-0.21)
Italy	4836 (1107-7306)	8.56 (1.97-12.91)	4249 (1018-6539)	3.54 (0.83-5.4)	-1.54 (-2.91--0.15)
Jamaica	147 (39-243)	14.89 (3.99-24.52)	264 (69-447)	14.95 (3.88-25.51)	0.29 (0.04-0.55)
Japan	1644 (386-2620)	1.67 (0.39-2.67)	1200 (291-2021)	0.39 (0.1-0.63)	-2.68 (-4.14--1.19)
Jordan	98 (27-153)	18.45 (5.09-28.77)	303 (79-502)	11.58 (3.03-19.07)	-1.53 (-3.06-0.02)
Kazakhstan	265 (58-405)	3.38 (0.75-5.17)	398 (99-623)	3.94 (0.99-6.2)	-0.66 (-1--0.3)
Kenya	159 (24-296)	4.42 (0.68-8.3)	555 (123-1002)	5.35 (1.14-9.57)	1.61 (0.18-3.06)
Kiribati	6 (1-10)	29.25 (5.75-52.32)	15 (3-27)	40.64 (9.07-71.94)	1.37 (0.48-2.26)
Kuwait	11 (2-19)	5.77 (1.02-9.87)	47 (11-79)	4.93 (1.12-8.53)	-0.16 (-1.61-1.3)
Kyrgyzstan	41 (9-63)	2.31 (0.49-3.54)	87 (19-135)	3.23 (0.72-5.01)	-0.12 (-0.68-0.45)
Lao People's Democratic Republic	87 (-2-170)	8.43 (-0.19-16.47)	90 (12-174)	4.07 (0.55-7.93)	-2.32 (-3.27--1.35)

Latvia	60 (14-88)	2.56 (0.58-3.76)	141 (31-219)	4.96 (1.09-7.72)	2.79 (1.61-3.98)
Lebanon	85 (21-136)	8.32 (2.04-13.25)	199 (48-330)	5.72 (1.37-9.45)	-0.29 (-0.58-0.01)
Lesotho	49 (6-92)	9.5 (1.09-18.23)	151 (17-280)	24.26 (2.95-45.25)	4.54 (3.74-5.35)
Liberia	32 (4-56)	7.04 (0.88-12.54)	102 (16-185)	11.9 (1.78-21.36)	0.99 (-0.42-2.42)
Libya	31 (8-53)	3.74 (0.92-6.32)	148 (42-250)	6.27 (1.78-10.61)	3.47 (2.47-4.48)
Lithuania	42 (10-61)	1.46 (0.35-2.17)	122 (25-194)	3 (0.63-4.74)	2.4 (1.18-3.64)
Luxembourg	14 (3-22)	4.09 (0.82-6.32)	13 (3-21)	1.81 (0.37-2.86)	-2.04 (-2.97--1.1)
Madagascar	175 (9-319)	7.93 (0.38-14.37)	407 (21-783)	8.53 (0.31-16.09)	-0.08 (-1.6-1.45)
Malawi	118 (14-210)	6.84 (0.78-12.36)	236 (34-445)	6.74 (0.89-12.5)	-0.5 (-1.9-0.93)
Malaysia	252 (40-428)	5.62 (0.92-9.55)	450 (109-752)	3.45 (0.81-5.82)	-0.85 (-1.6--0.1)
Maldives	3 (1-5)	8.19 (2.14-13.66)	4 (1-7)	2.98 (0.73-5.08)	-2.45 (-3.61--1.27)
Mali	193 (35-325)	11.47 (1.99-19.8)	486 (76-852)	13.64 (2.1-23.7)	0.18 (-1.31-1.7)
Malta	28 (8-42)	12.01 (3.26-17.84)	38 (11-57)	5.82 (1.66-8.7)	-1.2 (-2.14--0.25)
Marshall Islands	2 (0-4)	31.56 (3.17-58.48)	9 (1-17)	58.32 (7.46-109.67)	2.8 (1.49-4.12)
Mauritania	59 (7-98)	12.38 (1.56-20.81)	137 (25-242)	14.82 (2.68-26.12)	0.16 (-1.01-1.35)
Mauritius	36 (7-59)	9.29 (1.79-15.28)	182 (46-299)	18.09 (4.57-29.69)	4.84 (4.1-5.58)
Mexico	3352 (694-5536)	16.8 (3.42-27.74)	9020 (2104-15185)	13.67 (3.14-23.06)	0.42 (-0.25-1.1)
Micronesia (Federated States of)	5 (1-10)	22.4 (2.36-40.7)	11 (2-20)	31.56 (4.51-56.81)	1.89 (0.99-2.8)
Monaco	1 (0-1)	1.3 (0.36-2.16)	1 (0-1)	1.18 (0.31-1.93)	-0.21 (-1.87-1.48)
Mongolia	14 (2-22)	2.47 (0.36-4)	41 (7-63)	3.31 (0.56-5.14)	1.59 (0.76-2.43)
Montenegro	14 (3-21)	3.97 (0.82-6.18)	33 (8-54)	6.1 (1.41-9.99)	2.27 (1.69-2.84)

Morocco	219 (56-367)	3.33 (0.85-5.58)	1145 (298-1934)	6.99 (1.81-11.72)	3.87 (3.21-4.53)
Mozambique	271 (24-472)	10.18 (0.82-17.56)	508 (69-896)	10.01 (1.23-17.89)	-0.14 (-1.56-1.29)
Myanmar	1070 (131-1961)	9.27 (1.09-17.03)	1879 (316-3428)	7.22 (1.25-13.22)	-0.34 (-0.88-0.19)
Namibia	69 (6-119)	21.57 (1.87-37.48)	106 (11-203)	15.33 (1.64-29.43)	-1.41 (-2.43--0.38)
Nauru	0 (0-1)	25.57 (4.41-43.8)	1 (0-2)	34.11 (6.73-60.12)	1.51 (0.43-2.59)
Nepal	198 (26-341)	5.01 (0.67-8.72)	707 (128-1256)	6.55 (1.2-11.67)	1.81 (0.8-2.83)
Netherlands	828 (167-1300)	6.37 (1.29-9.96)	602 (139-946)	2.47 (0.58-3.85)	-2.33 (-3.39--1.27)
New Zealand	70 (12-115)	3.18 (0.56-5.23)	104 (20-167)	2.11 (0.41-3.34)	-1.42 (-2.11--0.72)
Nicaragua	53 (2-95)	6.75 (0.26-11.94)	212 (24-391)	8.19 (0.89-15.07)	1.67 (0.78-2.57)
Niger	59 (10-105)	5.29 (0.98-9.37)	262 (54-485)	7.6 (1.53-14.23)	1.07 (-0.69-2.86)
Nigeria	1277 (319-2218)	6.53 (1.64-11.33)	2956 (747-5243)	7.36 (1.94-12.85)	-0.14 (-1.55-1.29)
Niue	0 (0-0)	18.87 (2.71-33.02)	0 (0-1)	28.28 (5.02-50.47)	1.57 (1.14-2.01)
North Macedonia	70 (19-110)	7.75 (2.08-12.11)	193 (54-307)	12.11 (3.42-19.16)	2.39 (2-2.78)
Northern Mariana Islands	1 (0-2)	15.65 (3.51-27.16)	3 (1-5)	13.16 (3.1-22.12)	1.8 (0.57-3.05)
Norway	110 (26-167)	2.34 (0.56-3.54)	118 (27-182)	1.66 (0.37-2.53)	-0.98 (-2.25-0.3)
Oman	22 (4-40)	8.04 (1.46-14.23)	49 (9-87)	6.85 (1.26-12.24)	0.15 (-1.26-1.58)
Pakistan	2492 (515-4171)	11.46 (2.34-19.16)	9362 (2161-15263)	19.39 (4.45-31.37)	1.4 (0.16-2.65)
Palau	1 (0-2)	18.61 (2.71-33.08)	2 (1-4)	27.58 (5.85-48.73)	2.46 (1.96-2.96)
Palestine	59 (11-102)	14.22 (2.63-24.62)	127 (34-206)	12.11 (3.27-19.76)	-0.13 (-1.46-1.22)
Panama	33 (3-61)	4.63 (0.38-8.44)	118 (16-220)	4.98 (0.66-9.27)	1.03 (0.62-1.43)

Papua New Guinea	112 (22-202)	13.5 (2.61-23.84)	349 (63-611)	15.53 (2.88-26.61)	0.59 (-0.62-1.81)
Paraguay	71 (3-131)	6.3 (0.28-11.68)	351 (23-658)	11.7 (0.77-21.99)	2.96 (2.34-3.58)
Peru	120 (26-211)	2.05 (0.43-3.61)	411 (108-787)	2.34 (0.61-4.49)	1.1 (0.55-1.66)
Philippines	520 (99-926)	4.14 (0.79-7.4)	2657 (553-4665)	6.36 (1.33-11.12)	2.64 (1.77-3.51)
Poland	1331 (275-2119)	4.97 (1.03-7.9)	1862 (405-2917)	3.78 (0.83-5.92)	0.36 (-0.54-1.28)
Portugal	538 (101-886)	6.59 (1.25-10.76)	766 (141-1249)	3.92 (0.73-6.35)	-0.8 (-1.96-0.38)
Puerto Rico	255 (28-435)	13.58 (1.45-23.18)	331 (39-584)	6.76 (0.92-11.99)	-1.18 (-1.97--0.39)
Qatar	5 (1-8)	19.85 (5.61-33.35)	38 (11-63)	21.26 (6.23-35.4)	0.7 (-1.7-3.17)
Republic of Korea	516 (99-877)	3.25 (0.61-5.54)	1091 (201-1853)	1.93 (0.35-3.26)	-0.55 (-1.35-0.25)
Republic of Moldova	56 (12-89)	2.12 (0.45-3.36)	88 (19-141)	2.4 (0.52-3.86)	0.12 (-0.71-0.95)
Romania	352 (82-558)	2.29 (0.53-3.61)	527 (114-831)	2.22 (0.49-3.52)	1.07 (0.2-1.94)
Russian Federation	2250 (481-3371)	1.84 (0.39-2.75)	10389 (2389-15916)	6.35 (1.46-9.75)	3.31 (1.95-4.68)
Rwanda	168 (4-334)	12.24 (0.19-24.39)	177 (29-359)	6.03 (0.9-12.44)	-3.4 (-4.78--2)
Saint Kitts and Nevis	5 (1-8)	21.04 (4.63-34.7)	4 (1-6)	11.23 (2.38-19.02)	-1.48 (-1.72--1.24)
Saint Lucia	14 (2-23)	29.88 (4.57-50.16)	19 (3-33)	14.84 (2.45-25.42)	-2.22 (-2.68--1.75)
Saint Vincent and the Grenadines	13 (2-21)	32.32 (5.37-52.74)	10 (3-17)	15.14 (3.84-25.33)	-2.05 (-2.4--1.7)
Samoa	9 (1-17)	22.84 (1.51-40.61)	25 (3-44)	36.03 (4.93-62.51)	2.03 (1.31-2.76)
San Marino	1 (0-1)	2.89 (0.64-4.67)	1 (0-1)	1.4 (0.36-2.36)	-1.23 (-2.5-0.07)
Sao Tome and Principe	1 (0-2)	2.9 (0.49-4.96)	2 (0-3)	3.43 (0.73-5.69)	0.16 (-0.85-1.19)
Saudi Arabia	122 (34-200)	5.4 (1.51-8.77)	518 (181-842)	7.21 (2.5-11.81)	1.35 (-0.03-2.74)
Senegal	116 (25-200)	8.27 (1.76-14.52)	421 (90-711)	11.99 (2.52-20.37)	1.68 (0.41-2.96)

Serbia	351 (88-542)	6.66 (1.74-10.39)	624 (163-1006)	6.12 (1.63-9.86)	0.72 (-0.03-1.48)
Seychelles	1 (0-1)	2.06 (0.21-3.7)	2 (0-3)	2.69 (0.44-5.01)	1.79 (1.63-1.96)
Sierra Leone	37 (7-64)	4.18 (0.75-7.24)	117 (22-207)	7.14 (1.29-12.58)	1.62 (0.33-2.93)
Singapore	39 (5-69)	3.5 (0.49-6.22)	18 (3-31)	0.39 (0.06-0.66)	-5.6 (-6.39--4.81)
Slovakia	133 (28-213)	3.72 (0.78-5.96)	167 (38-272)	2.8 (0.63-4.55)	-0.08 (-0.78-0.63)
Slovenia	59 (11-95)	3.75 (0.71-6.06)	73 (16-117)	2.34 (0.52-3.74)	-2.28 (-3.4--1.14)
Solomon Islands	13 (1-25)	24.02 (0.96-44.39)	49 (1-91)	30.29 (0.42-55.66)	1.36 (0.1-2.63)
Somalia	155 (6-293)	14.61 (0.49-27.73)	484 (38-898)	16.88 (1.15-30.36)	0.37 (-1.31-2.09)
South Africa	1041 (147-1800)	9.16 (1.29-15.94)	4630 (797-7854)	18.28 (3.13-31.03)	3.44 (2.84-4.05)
South Sudan	71 (11-134)	7.45 (1.08-13.91)	152 (30-278)	9.69 (1.98-17.54)	0.49 (-1.19-2.19)
Spain	2482 (580-3823)	7.32 (1.71-11.27)	1913 (448-3057)	2.2 (0.52-3.5)	-2.79 (-4.04--1.52)
Sri Lanka	241 (39-425)	5.62 (0.87-9.92)	913 (216-1653)	6.23 (1.45-11.36)	2.41 (1.9-2.92)
Sudan	143 (34-250)	3.51 (0.84-6.06)	410 (102-753)	5.12 (1.27-9.19)	1.21 (-0.13-2.56)
Suriname	8 (2-13)	6.22 (1.7-10.25)	20 (6-36)	6.01 (1.78-10.6)	1.16 (0.77-1.55)
Sweden	310 (68-479)	3.04 (0.67-4.7)	382 (90-607)	2.42 (0.57-3.81)	-0.2 (-1.58-1.21)
Switzerland	374 (72-581)	5.15 (1-7.95)	233 (48-374)	1.66 (0.34-2.62)	-3.35 (-4.61--2.08)
Syrian Arab Republic	110 (29-183)	5.15 (1.38-8.49)	272 (73-466)	5.66 (1.49-9.42)	0.8 (-0.69-2.3)
Taiwan (Province of China)	534 (56-958)	7.87 (0.84-14.12)	1187 (146-2074)	4.89 (0.61-8.51)	-0.86 (-1.48--0.23)
Tajikistan	60 (14-94)	3.94 (0.94-6.16)	150 (37-248)	5.19 (1.31-8.6)	0.61 (-0.37-1.59)
Thailand	501 (69-923)	2.82 (0.39-5.22)	1614 (423-2915)	2.69 (0.71-4.85)	0.99 (0.55-1.43)

Timor-Leste	6 (0-11)	4.7 (0.11-8.88)	20 (1-38)	4.95 (0.28-9.44)	1.26 (0.12-2.41)
Togo	35 (6-61)	6.41 (0.97-11.34)	169 (27-303)	9.59 (1.49-17.35)	2.02 (0.52-3.54)
Tokelau	0 (0-0)	22.15 (2.52-40.88)	0 (0-0)	22.68 (3.59-40.71)	0.6 (0.37-0.83)
Tonga	7 (1-12)	25.78 (3.81-45.44)	12 (2-22)	28.52 (3.96-52.22)	1.06 (0.52-1.59)
Trinidad and Tobago	138 (36-223)	33.03 (8.64-53.28)	234 (61-401)	22.7 (5.95-38.79)	-0.41 (-0.74--0.09)
Tunisia	53 (14-90)	2.53 (0.67-4.24)	257 (69-443)	3.88 (1.03-6.71)	2.86 (2.34-3.39)
T眉rkiye	1692 (518-2716)	10.37 (3.16-16.63)	3591 (1111-5654)	7.5 (2.33-11.84)	0.86 (0.33-1.39)
Turkmenistan	42 (9-65)	3.79 (0.81-5.86)	211 (53-345)	9.13 (2.26-15)	3.07 (2.41-3.74)
Tuvalu	1 (0-2)	25.61 (2.49-45.31)	1 (0-3)	28.27 (4.2-50.8)	0.68 (0.41-0.96)
Uganda	177 (-1-358)	6.35 (-0.08-12.78)	431 (34-854)	6.27 (0.39-12.55)	-0.64 (-2.27-1.03)
Ukraine	595 (118-941)	1.27 (0.25-2.02)	475 (109-865)	0.97 (0.22-1.78)	-2.07 (-2.93--1.19)
United Arab Emirates	20 (5-32)	15.92 (4.17-25.57)	75 (19-120)	27.99 (7.26-44.92)	1.81 (-1.08-4.78)
United Kingdom	1854 (433-2844)	3.1 (0.72-4.75)	1223 (305-1893)	1.37 (0.35-2.12)	-2.09 (-3.21--0.96)
United Republic of Tanza-nia	278 (39-489)	5.84 (0.77-10.45)	583 (101-1057)	5.02 (0.86-9.08)	-0.78 (-2.11-0.56)
United States of America	9886 (2161-15445)	5.1 (1.12-7.93)	12172 (2985-18710)	3.61 (0.89-5.51)	-1.41 (-2.18--0.62)
United States Virgin Is-lands	4 (1-6)	9.37 (2.13-15.89)	5 (1-9)	4.86 (1.19-8.59)	-0.37 (-0.91-0.17)
Uruguay	162 (35-248)	6.93 (1.51-10.58)	230 (60-339)	6.13 (1.63-9)	0.15 (-0.62-0.93)
Uzbekistan	227 (54-343)	3.43 (0.82-5.19)	1401 (313-2244)	9.75 (2.21-15.51)	3.64 (2.98-4.31)
Vanuatu	4 (0-8)	16.14 (1.55-29.68)	18 (2-31)	21.83 (2.56-38.37)	1.61 (0.4-2.84)
Venezuela (Bolivarian Republic of)	291 (52-502)	6 (1.07-10.39)	1122 (216-2023)	6.98 (1.34-12.62)	1.82 (1.14-2.51)

Viet Nam	856 (82-1558)	3.82 (0.36-6.97)	2343 (91-4568)	4.45 (0.18-8.61)	1.71 (1.31-2.1)
Yemen	81 (21-149)	3.56 (0.92-6.48)	290 (67-532)	4.64 (1.09-8.37)	1.01 (-0.45-2.5)
Zambia	103 (11-178)	8.88 (0.88-15.5)	255 (33-466)	8.49 (1-15.69)	-0.55 (-2.21-1.13)
Zimbabwe	217 (3-388)	11.82 (0.13-21.17)	788 (21-1490)	22.67 (0.51-42.3)	3.49 (2.07-4.93)

Table S2. The number of DALYs cases and the age-standardized DALYs rates attributable to in 1990 and 2021, and its trends from 1990 to 2021 across countries and territories.

Characterist-ics	1990		2021		1990–2021
	**Number of DALYs cases(95% UI)**	**The age-standardized DALYs rate/100000(95% UI)**	**Number of DALYs cases(95% UI)**	**The age-standardized DALYs rate/100000(95% UI)**	**EAPC(95% CI)**
Afghanistan	16345 (1978-28419)	466.98 (57.05-810.67)	56240 (7583-98447)	911.5 (124.73-1599.86)	1.33 (0.28-2.4)
Albania	1303 (401-2062)	121.06 (36.95-191.79)	3957 (1139-6357)	178.25 (52.12-283.72)	3.04 (2.62-3.45)
Algeria	9437 (2537-15631)	152.76 (41.17-256.3)	57720 (17507-97175)	312.02 (93.62-525.97)	3.42 (2.77-4.08)
American Samoa	69 (14-117)	561.79 (109.43-960.95)	258 (57-449)	1031.15 (223.46-1797.06)	3.04 (2.46-3.63)
Andorra	30 (8-48)	107.1 (27.41-172.05)	112 (30-186)	154.3 (41.38-254.63)	2.02 (1.52-2.53)
Angola	6141 (598-10834)	290.7 (25.95-511.02)	16570 (3553-28617)	221.83 (45.09-385.29)	-1.44 (-2.63--0.23)
Antigua and Barbuda	136 (33-222)	463.19 (110.14-755.86)	293 (73-499)	511.23 (126.72-867.28)	0.46 (0.25-0.66)
Argentina	49170 (12000-76297)	273.78 (67.1-424.79)	86439 (22528-141369)	286.57 (75.49-466.85)	0.21 (-0.02-0.45)
Armenia	3827 (972-6139)	238.45 (61.11-382.72)	6142 (1521-10129)	254.57 (63.45-414.68)	0.69 (0.09-1.29)
Australia	12776 (2464-20714)	119.19 (23.32-193.14)	31119 (6411-52824)	137.86 (28.98-231.9)	0.87 (0.3-1.43)
Austria	8189 (1319-13432)	109.79 (18.2-180.56)	10733 (2396-18050)	114.81 (26.5-192.4)	0.69 (-0.23-1.62)

Azerbaijan	4958 (1427-7735)	164.07 (46.85-255.74)	21288 (5931-34006)	347.67 (98-558.22)	2.8 (2.52-3.08)
Bahamas	385 (64-654)	427.04 (69.9-729.53)	988 (207-1694)	426.54 (89.29-731.25)	0.54 (0.24-0.84)
Bahrain	493 (140-786)	629.98 (173.67-1010.51)	3262 (936-5345)	875.5 (238.29-1422.1)	1.56 (0.41-2.73)
Bangladesh	52938 (3971-93394)	242.42 (15.56-428.3)	206054 (34681-352669)	307.06 (50.6-527.33)	1.86 (0.89-2.85)
Barbados	992 (184-1656)	617.12 (118.88-1025.92)	1357 (300-2303)	507.63 (117.98-858.24)	-0.23 (-0.73-0.27)
Belarus	8203 (1888-13198)	102.25 (23.49-164.01)	11248 (2534-18574)	123.11 (28.03-201.15)	0.19 (-0.5-0.88)
Belgium	12443 (2985-19727)	142.45 (35.46-225.91)	18411 (4660-30559)	173.82 (45.75-287.13)	0.55 (-0.23-1.35)
Belize	145 (29-250)	304.92 (61.08-525.12)	590 (150-1026)	358.5 (90.64-632.24)	1.03 (0.26-1.81)
Benin	1891 (437-3155)	174.53 (39.79-292.25)	8392 (2109-14132)	273 (66.26-463.29)	1.36 (0.21-2.52)
Bermuda	76 (10-134)	214.5 (27.38-378.81)	135 (16-243)	205.19 (25.9-369)	0.62 (0.02-1.22)
Bhutan	226 (39-382)	173.41 (29.24-295.56)	685 (155-1169)	223.31 (49.78-382.8)	1.75 (1.03-2.47)
Bolivia (Plurinational State of)	5265 (869-9035)	298.13 (48.36-513.3)	15771 (2345-28952)	319.19 (46.89-585.31)	1.05 (0.46-1.65)
Bosnia and Herzegovina	4257 (1095-6828)	182.53 (46.46-290.75)	11306 (3393-18896)	327.13 (98.53-546.83)	3.23 (2.55-3.91)
Botswana	1088 (100-2035)	354.57 (31.65-654.09)	3137 (417-5499)	376.97 (48.08-663.13)	1.71 (0.75-2.67)
Brazil	124101 (18508-211250)	249.6 (36.51-427.48)	364889 (58089-626883)	263.47 (41.99-452.34)	1.16 (0.86-1.46)
Brunei Darussalam	261 (48-439)	502.18 (89.7-862.56)	908 (220-1546)	470.76 (111.03-811.12)	1.01 (0.14-1.88)
Bulgaria	17651 (4754-28065)	261 (70.75-413.27)	23864 (5621-39763)	312.54 (77.58-510.74)	1.03 (0.15-1.91)
Burkina Faso	5438 (802-9216)	239.1 (34.89-405.07)	15463 (2442-25596)	291.29 (44.62-486.81)	0.62 (-0.47-1.72)
Burundi	3339 (375-6066)	255.95 (27.51-470.19)	6051 (1064-10491)	242.13 (39.48-423.2)	-1.24 (-2.49-0.02)

Cabo Verde	130 (25-225)	100.48 (19.24-173.78)	638 (134-1095)	247.22 (51.26-426.48)	3.28 (2.81-3.74)
Cambodia	4003 (248-7446)	147.06 (8.49-273.78)	15423 (1426-28995)	209.38 (19.01-389.12)	1.9 (1.22-2.58)
Cameroon	3988 (886-7050)	165.83 (36.61-289.75)	17653 (4001-30429)	243.87 (54.17-429.67)	1.17 (0.05-2.31)
Canada	18316 (3354-30350)	100.19 (18.22-165.62)	55327 (12134-93948)	153.04 (33.5-258.4)	1.23 (0.53-1.93)
Central African Republic	2826 (89-5029)	430.45 (9.57-774.31)	7625 (204-13614)	557.68 (12.9-1016.71)	0.87 (-0.21-1.96)
Chad	4328 (670-7139)	292.2 (44.56-483.58)	15997 (2865-25959)	549.05 (96.96-894.29)	1.42 (0.09-2.77)
Chile	12659 (3695-19266)	227.63 (66.25-346.77)	38539 (10411-64127)	277.26 (75.48-458.58)	1.58 (1.29-1.88)
China	388007 (47005-702839)	85.93 (10.44-156.09)	1267470 (156578-2309721)	121.98 (15.61-221.49)	2.16 (1.85-2.47)
Colombia	15839 (2784-27975)	158.84 (26.77-282.79)	54318 (12965-97104)	182.46 (43.98-325.29)	0.98 (0.58-1.37)
Comoros	286 (7-539)	271.98 (4.64-517.37)	850 (63-1555)	311.15 (20.84-573.76)	1.15 (0.36-1.96)
Congo	2252 (106-4143)	368.23 (15.89-675.23)	6692 (552-12190)	431.51 (32-773.61)	0.53 (-0.4-1.46)
Cook Islands	58 (12-99)	946.7 (195.06-1612.23)	113 (26-198)	890.82 (206.06-1558.53)	0.93 (0.63-1.24)
Costa Rica	1948 (382-3357)	204.04 (38.72-356.48)	7641 (2159-13205)	258.56 (73.06-445.15)	1.52 (1.1-1.95)
C么te d'Ivoire	3517 (748-5780)	174.47 (36.05-287.84)	18108 (4250-30728)	303.92 (69.89-522.85)	2.61 (1.28-3.96)
Croatia	4346 (1167-7311)	121.21 (33.01-201.88)	8200 (2053-14147)	170.04 (44.78-292.93)	1.65 (0.8-2.52)
Cuba	11295 (2620-19208)	217.01 (50.03-368.43)	17135 (4674-30577)	184 (50.91-324.52)	0.38 (-0.11-0.88)
Cyprus	2192 (573-3384)	544.05 (144.5-852.76)	2877 (748-4578)	275.23 (72.8-435.88)	-2.08 (-2.43--1.73)
Czechia	13513 (2946-22223)	168.57 (36.79-278.69)	28175 (6543-48247)	243.96 (57.88-419.83)	2.59 (1.76-3.43)
Democratic People's Republic of Korea	5725 (904-10335)	56.37 (8.93-101.13)	16600 (3203-29883)	89.46 (17.33-160.77)	2.29 (2.03-2.55)

Democratic Republic of the Congo	23360 (1198-43815)	264.12 (11.03-497.45)	76095 (3032-138155)	357.02 (9.92-657.5)	0.97 (-0.08-2.03)
Denmark	3763 (693-6092)	86.66 (16.29-139.31)	7277 (1781-12027)	127.2 (32.26-210.57)	1.44 (0.63-2.26)
Djibouti	160 (32-278)	232.36 (45.26-393.14)	943 (208-1632)	301 (64.7-520.98)	1.49 (0.31-2.7)
Dominica	216 (61-351)	618.42 (176.98-1003.09)	327 (94-525)	757.22 (219.8-1209.67)	0.85 (0.65-1.06)
Dominican Republic	2826 (742-4868)	139.04 (36.38-239.33)	12013 (2867-21285)	225.88 (53.62-400.14)	2.72 (2.26-3.18)
Ecuador	5808 (1186-10064)	206.37 (40.7-359.15)	25211 (4786-44405)	290.58 (55.02-511.78)	1.87 (1.33-2.4)
Egypt	18652 (4435-31108)	138.92 (33.62-231.03)	101453 (25141-176980)	342.8 (81.37-598.53)	4.04 (3.09-5.01)
El Salvador	2454 (511-4234)	147.85 (30.1-254.86)	10098 (2887-16817)	286.23 (82.12-477.28)	2.92 (2.51-3.33)
Equatorial Guinea	358 (18-670)	319.77 (14.74-599.37)	1063 (211-1896)	317.86 (60.57-570.03)	-0.26 (-1.33-0.83)
Eritrea	2124 (158-3965)	308.32 (19.63-568.46)	5794 (675-10395)	340.07 (35.96-613.82)	0.94 (-0.15-2.05)
Estonia	1280 (328-2074)	101.63 (26.04-165.39)	2764 (674-4587)	193.01 (46.16-317.7)	2.2 (1.37-3.04)
Eswatini	701 (49-1240)	439.94 (28.95-777.74)	2279 (127-4316)	671.89 (35.78-1274.05)	2.64 (1.43-3.86)
Ethiopia	55487 (7380-93329)	544.31 (69.47-913.44)	63997 (12081-107058)	275.46 (50.47-464.14)	-2.77 (-4.05--1.48)
Fiji	2888 (369-5030)	1424.49 (185.7-2484.07)	8251 (1415-14129)	1933.06 (334.48-3315.59)	1.79 (1.26-2.32)
Finland	4376 (882-7246)	105.38 (21.47-173.51)	8528 (2040-14340)	161.37 (40.18-267.81)	1.62 (0.85-2.39)
France	47121 (9670-73475)	95.84 (20.05-151.02)	80037 (19086-128887)	112.41 (28.28-184.1)	0.97 (0.1-1.85)
Gabon	857 (6-1639)	274.26 (1.22-523.19)	1904 (179-3583)	324.83 (29.45-605.46)	0.3 (-0.43-1.03)
Gambia	368 (57-621)	213.66 (30.66-364.68)	2053 (326-3642)	383.23 (59.36-682)	2.23 (0.98-3.49)
Georgia	5645 (1523-9025)	149.82 (40.7-239.33)	9993 (2794-16175)	305.81 (86.53-493.43)	3.45 (2.79-4.12)
Germany	104059 (22594-166333)	129.97 (28.32-208.21)	135094 (32578-220044)	145.34 (35.8-239.44)	0.35 (-0.66-1.37)

Ghana	6784 (1692-11038)	193.34 (47.3-315.8)	26933 (6967-45370)	264.87 (67.54-444.6)	1.46 (0.57-2.37)
Greece	11778 (3089-19117)	148.58 (39.45-241.94)	19575 (5007-32245)	188.87 (49.31-308.13)	1.07 (0.32-1.82)
Greenland	22 (5-34)	128.85 (28.77-201.83)	43 (12-70)	131.84 (35.36-213.58)	0.62 (0.26-0.99)
Grenada	290 (55-475)	718.42 (141.49-1174.18)	384 (97-651)	640.34 (160.96-1087.01)	0.31 (0.07-0.55)
Guam	141 (36-239)	355.27 (89.09-605.61)	320 (87-552)	311.22 (86.39-539.22)	0.86 (0.49-1.23)
Guatemala	2393 (617-4042)	120.46 (30.6-202.79)	27377 (7243-45051)	434.52 (113.83-714.65)	4.6 (3.72-5.48)
Guinea	2351 (494-4003)	137.67 (28.7-236.84)	7359 (1664-12958)	240.3 (52.86-429.12)	1.55 (0.61-2.5)
Guinea-Bissau	677 (36-1188)	306.78 (13.9-540.88)	2146 (161-3821)	484.26 (32.58-887.42)	1.5 (0.29-2.72)
Guyana	1234 (222-2152)	579.89 (101.43-1016.88)	2254 (625-3848)	619.06 (171.47-1060.4)	1.16 (0.57-1.74)
Haiti	12437 (1642-21843)	704.42 (90.09-1247.09)	35106 (2903-65319)	841.32 (60.94-1568.23)	0.89 (0.1-1.68)
Honduras	1454 (275-2551)	122.62 (22.16-215.17)	9467 (2458-16889)	252.14 (64.2-447.27)	3.02 (2.24-3.8)
Hungary	16143 (3698-26340)	189.98 (44.06-310.36)	26598 (6732-44118)	249.32 (63.37-409.13)	1.66 (0.81-2.52)
Iceland	138 (35-219)	92.06 (23.38-146.18)	338 (81-582)	129.94 (31.75-223.09)	1.43 (1.03-1.83)
India	276400 (58238-472746)	117.84 (24.36-202.44)	1120385 (281320-1896593)	177.93 (44.17-301.09)	2.13 (1.6-2.67)
Indonesia	55444 (4744-100071)	101.33 (8.54-183.57)	117496 (22621-208919)	90.43 (17.21-159.86)	0.08 (-0.43-0.58)
Iran (Islamic Republic of)	17479 (4256-28870)	139.66 (33.77-230.89)	111547 (32903-181165)	275.55 (80.79-448.94)	3.72 (3.02-4.42)
Iraq	13317 (3653-21602)	312.48 (84.89-504.46)	61165 (16824-103325)	458.17 (124.36-776.05)	1.85 (0.98-2.72)
Ireland	2554 (529-4109)	115.52 (24.07-186.82)	4267 (1042-7140)	112.43 (27.83-191.03)	0.35 (-0.06-0.77)
Israel	5902 (1854-9053)	228.46 (72.41-349.12)	15455 (4771-24486)	236.3 (73.76-376.81)	0.2 (-0.24-0.64)

Italy	121809 (28929-184636)	238.41 (57.52-363.77)	134393 (34060-212723)	181.8 (47.81-291.36)	-0.25 (-1.23-0.74)
Jamaica	4053 (1135-6554)	435.26 (122.95-702.87)	7755 (2196-12895)	478.19 (135.4-793.17)	0.54 (0.29-0.79)
Japan	90321 (23486-150647)	96.78 (25.4-160.83)	188820 (50202-331915)	131.23 (35.21-224.82)	1.71 (0.85-2.59)
Jordan	3458 (984-5552)	530.44 (151.82-845.97)	18403 (5302-30323)	492.67 (137.92-808.28)	0.3 (-0.82-1.44)
Kazakhstan	15594 (3720-25298)	198.28 (47.55-322.7)	36563 (9721-58860)	338.13 (89.9-542.84)	1.43 (1.26-1.6)
Kenya	5229 (824-9587)	120.31 (18.44-221.03)	19274 (4668-33632)	148.76 (34.83-260.27)	1.61 (0.44-2.8)
Kiribati	188 (40-327)	859.96 (177.77-1486.03)	546 (118-962)	1221.47 (259.93-2148.67)	1.57 (0.92-2.22)
Kuwait	690 (134-1191)	253.02 (45.86-438.27)	6704 (1699-11422)	388.37 (94.02-667.38)	2.45 (1.53-3.38)
Kyrgyzstan	2242 (517-3530)	125.23 (28.74-197.49)	6740 (1662-10894)	227.29 (56.63-366.14)	1.56 (1.15-1.97)
Lao People's Democratic Republic	2959 (-48-5795)	257.49 (-4.72-500.64)	4057 (559-7564)	157.73 (22.36-296.23)	-1.42 (-2.23--0.59)
Latvia	2892 (720-4471)	131.94 (32.92-204.44)	5428 (1302-8817)	250.09 (62.12-405.53)	2.42 (1.55-3.3)
Lebanon	3417 (915-5492)	297.37 (78.98-476.85)	11319 (3031-18791)	352.19 (94.45-584.14)	1.22 (1.02-1.41)
Lesotho	1282 (158-2358)	238.93 (28.76-441.13)	4415 (510-8092)	655.51 (77.54-1201.46)	4.78 (4.07-5.49)
Liberia	1074 (166-1846)	198.12 (29.34-346.06)	4208 (692-7268)	364.71 (55.78-635.15)	1.5 (0.35-2.65)
Libya	1539 (392-2501)	166.56 (42.05-269.08)	9775 (2723-16495)	335.38 (93.22-568.3)	3.9 (3.09-4.71)
Lithuania	2580 (657-4169)	95.93 (24.47-155.36)	5546 (1325-9031)	178.38 (43.38-288.58)	2.36 (1.57-3.15)
Luxembourg	407 (91-639)	130.87 (29.89-206.47)	754 (182-1239)	146.59 (36.4-241.85)	0.4 (-0.23-1.04)
Madagascar	5673 (335-10382)	214.33 (11.83-390.68)	16101 (1114-30018)	248 (13.01-455.2)	0.41 (-0.79-1.62)
Malawi	3581 (469-6235)	172.22 (21.71-306.26)	7536 (1245-13477)	176.04 (26.48-316.99)	-0.34 (-1.52-0.86)

Malaysia	9765 (1509-16908)	197.45 (30.86-344.5)	27524 (7620-48278)	186.87 (51.2-326.35)	0.67 (0.11-1.24)
Maldives	102 (26-174)	248.01 (63.7-418.77)	219 (58-380)	139.87 (36.39-243.82)	-0.98 (-1.93--0.01)
Mali	7881 (1390-13218)	376.75 (65.77-639.29)	23942 (4281-40256)	502.84 (85.63-857.79)	0.67 (-0.48-1.83)
Malta	680 (199-1002)	285.4 (83.94-421.72)	1300 (405-2039)	275.78 (87.4-426.67)	0.78 (0.04-1.52)
Marshall Islands	87 (9-157)	976.25 (102.16-1758.11)	380 (57-694)	1871.49 (259.16-3401.74)	3.17 (2.18-4.18)
Mauritania	1803 (237-2943)	342.32 (44.73-559.25)	4797 (983-8202)	430.24 (85.72-740.32)	0.44 (-0.55-1.44)
Mauritius	1330 (270-2231)	322.69 (65.06-543.77)	6055 (1515-10307)	611.42 (156.39-1036.73)	4.13 (3.61-4.66)
Mexico	127266 (28662-211413)	540.82 (117.75-904.14)	374378 (92051-638175)	534.91 (130.17-913.07)	0.85 (0.35-1.35)
Micronesia (Federated States of)	175 (21-309)	678.93 (79.72-1197.43)	438 (69-764)	1047.72 (158.43-1835.02)	2.41 (1.69-3.12)
Monaco	25 (7-40)	70.83 (21.7-113.58)	54 (16-90)	131.99 (38.88-219.2)	1.94 (0.9-2.98)
Mongolia	775 (130-1242)	130.6 (21.9-211.06)	3244 (653-5248)	219.99 (43.38-350.08)	2.55 (1.9-3.2)
Montenegro	694 (147-1148)	198.74 (42.5-328.58)	1635 (401-2663)	305.45 (76.13-495.36)	2.15 (1.62-2.67)
Morocco	12841 (3439-21020)	172.65 (45.85-282.35)	83587 (22911-143663)	456.87 (124.59-780.61)	4.39 (3.9-4.89)
Mozambique	8423 (774-14383)	261.71 (22.46-447.09)	18107 (2559-31016)	276.36 (36.02-471.89)	0.13 (-1.02-1.3)
Myanmar	36857 (4769-67306)	282.83 (35.69-517.65)	76027 (12980-134958)	268.01 (46.02-475.49)	0.28 (-0.12-0.68)
Namibia	1943 (173-3310)	542.84 (47.75-929.25)	3117 (354-5787)	393.52 (42.6-733.52)	-1.28 (-2.15--0.4)
Nauru	19 (4-33)	798.5 (153.79-1388.02)	42 (10-73)	1160.01 (258.47-2039.67)	1.66 (0.87-2.46)
Nepal	8630 (1269-14866)	174.86 (24.57-304.33)	33654 (6533-58428)	259.05 (50.38-449.46)	2.14 (1.39-2.89)
Netherlands	18694 (3944-29787)	158.98 (33.99-252.92)	21738 (5251-35326)	128.13 (31.49-209.93)	-0.48 (-1.24-0.29)
New Zealand	2944 (524-4862)	144.51 (26.06-237.24)	6593 (1301-10922)	162.69 (33.08-269.18)	0.69 (0.22-1.17)

Nicaragua	2416 (134-4329)	272.5 (13.47-493.23)	11258 (1600-20157)	397.77 (53.85-713.39)	2.16 (1.39-2.93)
Niger	2567 (501-4287)	178.43 (34.3-297.61)	13087 (2657-22592)	283.51 (57-486.58)	1.45 (0.08-2.85)
Nigeria	38624 (9712-63754)	180.51 (45.44-298.71)	114656 (28941-194752)	218.67 (55.58-373.15)	0.39 (-0.78-1.58)
Niue	7 (1-13)	592.4 (91.18-1055.94)	11 (2-19)	977.61 (194.45-1706.7)	1.93 (1.59-2.27)
North Macedonia	2762 (773-4441)	281.4 (78.59-449.75)	7888 (2407-12511)	451.98 (137.53-708.03)	2.45 (2.02-2.88)
Northern Mariana Islands	43 (10-71)	466.68 (104.64-800.6)	132 (35-220)	491.16 (126.39-815.84)	2.14 (1.37-2.92)
Norway	4095 (1041-6557)	114.76 (29.85-182.62)	5779 (1413-9304)	123.78 (31.17-200.83)	0.06 (-0.67-0.78)
Oman	772 (151-1318)	250.26 (48.6-428.95)	2597 (516-4737)	262.67 (49.82-474.9)	0.83 (-0.37-2.04)
Pakistan	86234 (18617-139429)	333.55 (70.87-540.59)	386231 (96794-625682)	617.19 (152.11-984.95)	2.02 (1.06-3)
Palau	31 (5-53)	588.5 (90.11-1016.13)	96 (22-171)	880.61 (199.1-1552.77)	2.52 (2.21-2.83)
Palestine	1697 (316-2896)	365.74 (68.03-622.78)	5117 (1439-8449)	387.3 (106.16-648.68)	0.71 (-0.4-1.83)
Panama	1346 (144-2473)	172.78 (17.7-320.58)	5502 (749-9959)	243.32 (33.42-440.8)	1.7 (1.37-2.02)
Papua New Guinea	4380 (845-7657)	430.37 (82.8-755.58)	17159 (3526-29456)	579.83 (117.77-991.94)	1.26 (0.36-2.16)
Paraguay	2575 (157-4674)	214.65 (12.68-392.75)	12123 (957-22352)	390.4 (29.67-722.68)	2.73 (2.18-3.28)
Peru	4522 (1070-7917)	70.71 (16.35-123.1)	17185 (4933-30409)	96.59 (27.72-170.8)	1.71 (1.24-2.18)
Philippines	20551 (4113-36420)	128.8 (25.49-230.55)	102572 (22482-181021)	222.16 (48.29-393.95)	2.75 (2.09-3.42)
Poland	60660 (13313-96549)	237.25 (52.2-375.73)	92344 (20700-150523)	234.04 (53.53-383.87)	0.87 (0.18-1.56)
Portugal	15267 (3180-25284)	196.69 (41.79-324.22)	29268 (5804-50389)	242.92 (51.28-406.79)	1.03 (0.2-1.87)
Puerto Rico	7937 (902-13896)	411.53 (46.82-721.28)	12190 (1720-22270)	352.32 (59.97-637.84)	0.02 (-0.54-0.59)

Qatar	211 (60-334)	570.83 (154.98-905.55)	2865 (902-4719)	783.45 (240.48-1287.87)	1.8 (0.11-3.52)
Republic of Korea	23428 (4810-40779)	128.95 (26.17-223.33)	98537 (18748-178725)	213.9 (41.8-387.15)	2.66 (2.16-3.16)
Republic of Moldova	3194 (745-5336)	120.36 (27.98-201.33)	6017 (1400-10431)	182.31 (42.96-315.6)	1.58 (1.06-2.1)
Romania	18543 (4600-31274)	119.86 (29.59-201.47)	32449 (8171-54836)	166.74 (43.52-274.69)	2 (1.31-2.7)
Russian Federation	130581 (30165-205008)	113.35 (26.14-177.88)	355040 (85045-559956)	242.01 (58.91-382.02)	2.23 (1.47-3)
Rwanda	5225 (211-10117)	318.22 (8.87-610.82)	5602 (1050-10594)	151.96 (26.12-294.2)	-3.4 (-4.52--2.27)
Saint Kitts and Nevis	130 (30-216)	635.27 (145.22-1054.6)	173 (40-294)	461.37 (108.14-782.56)	-0.32 (-0.55--0.09)
Saint Lucia	435 (69-743)	901.77 (142.89-1536.14)	767 (150-1355)	613.1 (120.72-1083.18)	-0.7 (-1.07--0.34)
Saint Vincent and the Grenadines	358 (64-586)	912.62 (164.03-1498.08)	408 (116-697)	589.14 (169.48-1012.25)	-0.91 (-1.22--0.6)
Samoa	328 (25-565)	733.85 (55.2-1267.38)	984 (139-1670)	1292.13 (181.01-2200.33)	2.37 (1.78-2.97)
San Marino	19 (5-30)	99.24 (25.66-158.21)	45 (12-77)	138.59 (38.17-233.06)	1.48 (0.7-2.26)
Sao Tome and Principe	47 (8-81)	133.44 (23.48-231.67)	135 (35-234)	207.08 (51.5-358.84)	1.42 (0.57-2.28)
Saudi Arabia	5205 (1526-8466)	192.44 (55.11-312.09)	38992 (14552-61780)	350.27 (130.24-559.99)	2.88 (1.89-3.88)
Senegal	4383 (951-7400)	261.73 (55.98-447.63)	17125 (3622-28665)	403.67 (85.42-682)	1.93 (0.91-2.96)
Serbia	14539 (3819-23240)	241.93 (65.24-384.26)	26161 (7177-43827)	296.29 (82.1-493.96)	1.25 (0.57-1.94)
Seychelles	26 (3-49)	82.08 (9.71-154.97)	96 (15-187)	158.52 (25.38-310.62)	3 (2.84-3.15)
Sierra Leone	1306 (249-2246)	127.91 (23.95-221.3)	5108 (1091-8818)	247.54 (48.96-434.87)	2.15 (1.1-3.21)
Singapore	1951 (277-3592)	152.63 (21.45-285.59)	6078 (1172-11481)	143.41 (28.15-269.28)	0.86 (0.58-1.14)
Slovakia	5531 (1231-9178)	160.78 (35.46-266.42)	10080 (2323-16916)	192.64 (44.69-319.7)	1.34 (0.74-1.94)
Slovenia	2501 (502-4164)	169.14 (34.77-280.51)	3915 (922-6470)	174.97 (41.63-286.35)	-0.06 (-0.89-0.77)

Solomon Islands	477 (22-872)	689.1 (30.34-1255.16)	1918 (63-3465)	949.61 (23.96-1721.22)	1.67 (0.7-2.65)
Somalia	5332 (219-9722)	387.39 (14.85-711.15)	17657 (1474-31392)	467.66 (35.19-831.96)	0.49 (-0.84-1.83)
South Africa	34042 (4909-57807)	277.33 (39.16-471.79)	146954 (25267-251674)	538.62 (91.98-920.58)	3.36 (2.89-3.84)
South Sudan	2130 (355-3823)	193.83 (31.64-353.76)	5504 (1108-9730)	265.94 (54.44-474.71)	0.97 (-0.42-2.38)
Spain	72883 (17962-116645)	234.15 (57.95-375.2)	106690 (26336-178925)	226.35 (57.62-379.91)	0.03 (-0.8-0.86)
Sri Lanka	8999 (1567-15965)	172.86 (28.67-308.67)	39638 (9738-70323)	264.12 (65.25-470.56)	2.9 (2.54-3.26)
Sudan	6571 (1679-10645)	140.68 (35.65-228.75)	26504 (7497-45555)	252.54 (70.62-434.65)	2.05 (0.96-3.16)
Suriname	321 (96-536)	231.42 (67.83-390.63)	1127 (347-1899)	328.14 (101.03-552.35)	2.18 (1.9-2.47)
Sweden	8902 (2082-14304)	112.88 (26.78-181.67)	14225 (3487-23183)	144.04 (36.32-234.78)	0.92 (0.05-1.8)
Switzerland	10201 (2163-16268)	171.96 (37.14-274.91)	16705 (4024-28064)	203.33 (49.16-346.17)	0.5 (-0.29-1.3)
Syrian Arab Republic	4894 (1408-8114)	185.95 (51.29-307.26)	18324 (5234-31188)	273.26 (77.51-460.33)	2.2 (1.18-3.24)
Taiwan (Province of China)	17676 (1973-31547)	232.55 (25.86-414.42)	48701 (6874-86700)	221.61 (32.27-389.71)	0.44 (-0.06-0.94)
Tajikistan	2427 (624-3818)	154.43 (39.64-242.22)	8960 (2429-14564)	259.63 (70.19-420.01)	1.79 (0.95-2.62)
Thailand	17766 (2835-31460)	89.34 (13.57-160.15)	68928 (19097-121448)	120.81 (34.03-211.82)	2.08 (1.7-2.46)
Timor-Leste	211 (7-394)	143.53 (3.44-272.18)	913 (58-1721)	207.85 (12.39-394.63)	2.04 (1.11-2.99)
Togo	1259 (223-2201)	181.86 (30.8-312.21)	6573 (1048-11426)	288.81 (44.69-501.12)	2.23 (1.07-3.4)
Tokelau	5 (1-9)	708.54 (92.95-1260.25)	6 (1-12)	863.1 (156.75-1528.9)	1.04 (0.86-1.22)
Tonga	236 (36-411)	787.38 (120.38-1375.29)	410 (61-747)	944.88 (140.06-1735.09)	1.11 (0.63-1.58)
Trinidad and Tobago	4407 (1171-7083)	998.03 (264.45-1607.24)	8348 (2317-14068)	842.56 (240.11-1409.68)	0.15 (-0.14-0.44)
Tunisia	3111 (871-5209)	122.74 (34.22-207.2)	19078 (5782-32432)	271.78 (82.62-461.09)	3.67 (3.3-4.05)

T眉rkiye	49738 (14823-79802)	275.33 (82.44-440.71)	156576 (52183-255125)	313.44 (105.13-510.03)	1.85 (1.42-2.27)
Turkmenistan	1675 (390-2654)	145.44 (33.92-229.5)	9161 (2288-14640)	376.36 (93.45-601.13)	3.58 (3.03-4.14)
Tuvalu	30 (3-53)	755.75 (77.73-1321.06)	52 (8-90)	916.33 (134.24-1592.91)	0.9 (0.74-1.06)
Uganda	5123 (70-10044)	155.95 (0.51-307.76)	13607 (1537-25309)	158.16 (15.45-298.52)	-0.47 (-1.86-0.94)
Ukraine	39822 (8286-64672)	92.55 (19.28-150.1)	53055 (12144-90311)	124.74 (28.36-210.97)	0.68 (0-1.36)
United Arab Emirates	796 (210-1265)	503.94 (130.49-805.19)	5683 (1695-9496)	666.18 (190.57-1070.5)	1.62 (-0.39-3.67)
United Kingdom	58962 (14680-92719)	121.74 (30.61-190.46)	105205 (28857-174373)	191.44 (53.87-317.62)	1.35 (0.68-2.02)
United Republic of Tanzania	8264 (1252-14248)	146.26 (20.44-255.45)	20097 (4127-35817)	140.92 (26.35-247.37)	-0.34 (-1.45-0.77)
United States of America	338972 (78184-535037)	197.35 (45.99-310.89)	878371 (236866-1418563)	307.63 (84.32-497.65)	1.55 (1.01-2.09)
United States Virgin Islands	169 (40-284)	354.87 (84.74-597.89)	324 (88-562)	386.4 (107.31-672.31)	1.53 (1.05-2.02)
Uruguay	4481 (1070-6865)	204.78 (49.32-314.35)	8715 (2510-13980)	294.04 (85.59-471.33)	1.58 (1.03-2.14)
Uzbekistan	10380 (2676-16406)	153.96 (39.63-243.54)	69515 (16771-111416)	441.36 (106.9-699.99)	4.01 (3.45-4.57)
Vanuatu	149 (14-269)	469.02 (43.68-845.08)	727 (84-1265)	731.14 (84.54-1268.29)	2.06 (1.13-2.99)
Venezuela (Bolivarian Republic of)	11420 (2278-19681)	208.38 (40.45-361.73)	46380 (9791-79843)	284 (60.04-487.05)	2.26 (1.72-2.8)
Viet Nam	23796 (2308-42742)	102.32 (9.95-183.41)	78433 (2549-152312)	141.01 (4.96-274.39)	2.23 (1.88-2.59)
Yemen	3697 (959-6223)	139.59 (35.96-237.15)	18272 (4437-30785)	229.51 (53.83-385.76)	1.82 (0.57-3.1)
Zambia	3624 (430-6327)	249.51 (27.6-435.08)	10854 (1413-19541)	268.1 (31.83-482.53)	-0.03 (-1.38-1.33)
Zimbabwe	6250 (135-11014)	303.62 (5.68-535.83)	25483 (718-46849)	622.54 (16.48-1154.27)	3.75 (2.51-5)
